# Substantial genome synteny preservation among woody angiosperm species: comparative genomics of Chinese chestnut (*Castanea mollissima*) and plant reference genomes

**DOI:** 10.1186/s12864-015-1942-1

**Published:** 2015-10-05

**Authors:** Margaret Staton, Tetyana Zhebentyayeva, Bode Olukolu, Guang Chen Fang, Dana Nelson, John E Carlson, Albert G Abbott

**Affiliations:** Department of Entomology and Plant Pathology, University of Tennessee Institute of Agriculture, Knoxville, TN USA; Department of Genetics and Biochemistry, Clemson University, Clemson, SC USA; Department of Plant Pathology, North Carolina State University, Raleigh, NC USA; USDA/APHIS/BRS, Raleigh, NC USA; Southern Institute of Forest Genetics, Southern Research Station, U.S. Forest Service, Saucier, MS USA; The School of Forest Resources and The Huck Institutes of the Life Sciences, Pennsylvania State University, University Park, PA USA; Department of Forestry, Forest Health Research and Education Center, Lexington, KY USA

**Keywords:** Chestnut, Comparative genomics, Blight resistance, Synteny, Plant genomics, Physical map, Genetic map

## Abstract

**Background:**

Chinese chestnut (*Castanea mollissima*) has emerged as a model species for the Fagaceae family with extensive genomic resources including a physical map, a dense genetic map and quantitative trait loci (QTLs) for chestnut blight resistance. These resources enable comparative genomics analyses relative to model plants. We assessed the degree of conservation between the chestnut genome and other well annotated and assembled plant genomic sequences, focusing on the QTL regions of most interest to the chestnut breeding community.

**Results:**

The integrated physical and genetic map of Chinese chestnut has been improved to now include 858 shared sequence-based markers. The utility of the integrated map has also been improved through the addition of 42,970 BAC (bacterial artificial chromosome) end sequences spanning over 26 million bases of the estimated 800 Mb chestnut genome. Synteny between chestnut and ten model plant species was conducted on a macro-syntenic scale using sequences from both individual probes and BAC end sequences across the chestnut physical map. Blocks of synteny with chestnut were found in all ten reference species, with the percent of the chestnut physical map that could be aligned ranging from 10 to 39 %.

The integrated genetic and physical map was utilized to identify BACs that spanned the three previously identified QTL regions conferring blight resistance. The clones were pooled and sequenced, yielding 396 sequence scaffolds covering 13.9 Mbp. Comparative genomic analysis on a microsytenic scale, using the QTL-associated genomic sequence, identified synteny from chestnut to other plant genomes ranging from 5.4 to 12.9 % of the genome sequences aligning.

**Conclusions:**

On both the macro- and micro-synteny levels, the peach, grape and poplar genomes were found to be the most structurally conserved with chestnut. Interestingly, these results did not strictly follow the expectation that decreased phylogenetic distance would correspond to increased levels of genome preservation, but rather suggest the additional influence of life-history traits on preservation of synteny. The regions of synteny that were detected provide an important tool for defining and cataloging genes in the QTL regions for advancing chestnut blight resistance research.

**Electronic supplementary material:**

The online version of this article (doi:10.1186/s12864-015-1942-1) contains supplementary material, which is available to authorized users.

## Background

Chestnuts are members of the Fagaceae family, comprised of about 900 species of trees and shrubs including other dominant Northern Hemisphere temperate forest trees such as oaks and beeches [1]. Many members of this family are subject to significant biotic stresses, particularly from fungal and fungal-like diseases. Examples include *Phytophthora cinnamomi,* an oomycete that attacks hundreds of plant species around the world including both oaks and chestnuts [2, 3], and *Phytophthora ramorum*, the cause of sudden oak death, a disease currently devastating oaks in western North America [4]. Rapid climate change and alien pathogen introduction have substantially increased the need for genetic tools to insure conservation and improvement of tree resources to meet future environmental challenges. A case in point, the American chestnut (*Castanea dentata*), was eliminated as a dominant eastern US forest tree species during the first half of the 20^th^ century due an introduced fungal pathogen (*Cryphonectria parasitica*) [5]. Utilizing the natural resistance of Chinese chestnut (*Castanea mollissima*) to the blight, chestnut breeding programs have for decades been working to produce a tree of primarily American chestnut genetic background with blight resistance [6]. To assist the breeding efforts and to enable future genetic research in chestnut, an extensive genomic tool development initiative for Chinese chestnut was undertaken, resulting in an integrated set of resources including transcriptome sequences [7], a dense genetic map [8], BAC libraries and a genetically anchored physical map [9]. These genomic tools create a powerful platform for trait mapping, applied breeding and comparative genomics.

Comparative genomics affords the opportunity to increase our understanding of the common underlying gene networks and molecular mechanisms of forest tree defense responses that may be conserved among plant species, especially close relatives suffering from similar diseases. However, in deciduous trees, we have limited knowledge of the degree of preservation of the structural organization of the genomes among either close or distantly related species. With the increase in genetic and genomic resources in some key forest and fruit trees species, we are now positioned to comparatively examine the extent of genome preservation at both low and high resolution among these species. These comparisons provide the opportunity to assess the ability to leverage the genomic information from highly characterized plant genomes for application in other less well-characterized species.

The physically mapped, genetically anchored EST sequence markers from the Chinese chestnut genetic map and the Chinese chestnut BAC end sequences (BES) spanning the physical map provide ideal substrates for genome comparisons with other tree species with highly characterized genomes at the sequence level, such as peach (*Prunus persica*) and poplar (*Populus trichocarpa*). Identified conserved syntenic blocks in these genomes could assist in candidate gene discovery when coupled with chestnut trait mapping efforts. Furthermore, these genomic comparisons provide a first low-resolution glimpse of the extent of genome change that has proceeded since the split of the families. However, to utilize comparative approaches with completely sequenced and assembled tree reference genomes as a means to identify potential candidate genes in marked intervals of less well sequence characterized species, we need to access the degree of preservation among tree genomes at a high resolution level, e.g. within 1 centiMorgan (cM) windows.

In order to gain both a low and a high resolution comparative picture of the extent genome change between Chestnut and other completely sequenced and assembled plant species genomes, we carried out comparative genome analyses utilizing BAC end sequences representing the physical map minimal tiling path and genetically anchored EST sequences in chestnut against completely sequenced and assembled genomes of angiosperm plant species of both phylogenetically close and distant relationships to chestnut. After completing this low-resolution comparative analysis, we were interested in assessing whether at high resolution the genome structural comparative relationships would be similar or different. Therefore, we carried out a comparative analysis between sequences of three regions of the Chinese chestnut genome that contain QTLs for chestnut blight resistance [8] with other plant genomes. These sequences were mapped to a number of completely sequenced plant genomes to examine the extent of genome preservation for these regions among diverse plant species. In addition to enabling us to evaluate the extent of preservation of the defined regions of the chestnut genome to other species, these sequences also provided genes to advance the study of the molecular underpinnings of blight resistance in Chinese chestnut as well as a set of markers for use in marker assisted breeding efforts.

In both the low and high resolution comparative analyses, we demonstrate that the peach genome is the best current reference model for chestnut, however surprisingly the grape genome is the second most highly similar genome in this study. Additionally, the comparative sequence analyses for the chestnut blight QTLs enabled us to define in high resolution the degree of gene preservation within the two chestnut QTLs that were previously found to overlap with mapped QTLs for powdery mildew resistance in peach, as reported by [8], suggesting that these regions might be important in generalized resistance to fungal pathogens. The combination of low and high resolution mapping of genomic sequence data provided in this report suggests that key high quality sequenced woody species genomes could serve as repositories of gene information for candidate gene studies in other related tree species with less well characterized genomes.

## Results

### Alignments of integrated physical and genetic map to reference genomes

Significant improvements have been made to the integrated physical and genetic map since their publication [9]. An additional 364 overgo probes have been successfully placed on the physical map, bringing the total overgo probes to 1,390. The total number of physical map contigs is 1300, a slight reduction from the previous version. The new probes were designed from Chinese Chestnut EST sequences that contain genetic map markers in order to create more links between the physical and genetic maps. This version of the physical map is available on the Hardwood Genomics Web as version 4 [10]. The new probes hybridized to an average of 16.6 BAC clones each, slightly lower than the previously reported average of 17.5. Because the BACs included in the physical map represent 18X genome equivalents, this suggests that the majority of probes are hybridizing to a single genomic location. Less than 3 % of probes (41) hybridized to more than 50 clones; those that did are likely repetitive sequences in the genome. More than 73 % of probes (1,024) hybridized to one or two assembled contigs in the physical map. This confirms that most unique genomic locations have been assembled into physical map contigs, yielding accurate links from a genetic map location to a physical map location.

Of the 1,390 overgo probes, 1,013 originated from an EST contig also used to develop one or more genetic map markers, thereby allowing an association from the overgo probe to a linkage group. Of these overgo probes, 858 are associated with both a linkage group and a cM position from the consensus genetic map. The latter set of markers was uploaded into the FPC software as framework markers and placed using the FPC ctg - > chr algorithm. This resulted in the placement of 342 physical map contigs to linkage group locations. The 342 anchored physical map contigs span an estimated 522 Mb and represent 41.9 % of the total physical map span. A further 174 contigs are anchored to a single genetic marker by only 1 BAC hybridization, and the BAC marker had stronger hybridization signals to a different physical map contig. These contigs are not considered anchored but are noted in the FPC file. The algorithm found 129 contigs to contain strong overgo links to more than one genetic map location, and these also were not considered anchored. These contigs may be incorrectly assembled or the probes may actually reside at multiple locations in the genome.

In addition to the use of overgo probes, the physical map has been enhanced through BAC-end sequencing. Forward and reverse end-sequencing was carried out on a subset of the clones in the map assembly, resulting in 42,970 sequences [GenBank:HN270092-HN275251, JY172573-187037]. Part of this BES subset was the 1,132 BACs spanning the QTL regions of the genome; the rest were selected based on physical map spacing, with an effort to obtain evenly spaced sequences spanning all contigs. The sequences include 26,873,995 bases. This represents 3.4 % of the chestnut genome, estimated to be 794 Mb [11]. The BAC library CMCMBb contained 19,064 of the BAC end sequences, of which 8,803 BACs had both uccessful forward and reverse sequences, creating a linked pair. The library CMCMBd had 23,872 BES and 11,187 successful BAC end sequence pairs.

The integrated physical and genetic map is a powerful genomic tool for targeted sequencing as well as comparison to other plant genomes. Using the BES spanning the physical map and the genetically-anchored EST-derived overgo probes, the software Symap was used to align the FPC contigs to the sequenced genomes of peach, strawberry, soybean, Medicago, poplar, papaya, Arabidopsis, Eucalyptus, grape and tomato. Peach and strawberry are members of the Rosales order, and they are the phylogenetically the closest examined relatives to the chestnut, which is in the Fagales order. Soybean, Medicago and poplar are members of the Fabidae/Rosid I clade, in orders more distant from Fagales. Arabidopsis, Eucalyptus and papaya fall into the sister Malvidae/Rosid II group. Grape is a core eudicot that is classified as an outgroup to all rosids. The furthest species from chestnut phylogenetically is tomato, a member of the asterids [12].

**Fig. 1:**
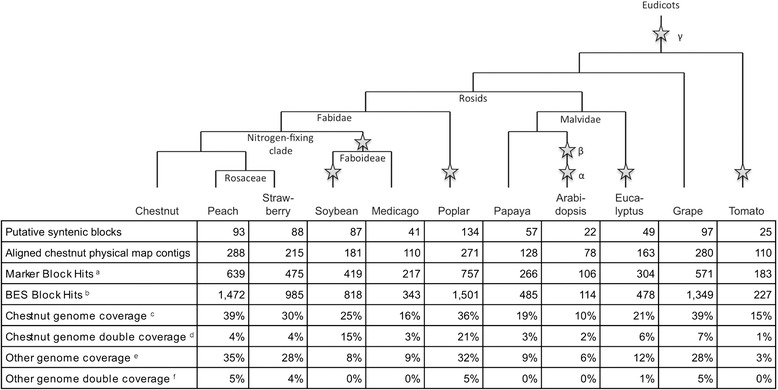
Alignments from chestnut to other plant genomes. Symap predicted alignments of the integrated chestnut genetic and physical map to the reference genomes of ten plant species with reference genome sequences. The species range broadly in phylogenetic position relative to chestnut; the relative phylogenetic position of the species is represented at the top of the table [12, 22, 23]. The locations of predicted whole genome duplication events (WGDs) during rosid evolution are marked with stars. All marked duplications were tetraploidizations except for the paleo hexaploidy event γ (gamma) which is ancestral to the rosid/asterid split [19, 20, 42] and a later triplication of the Solanum lineage, impacting the tomato genome [19]. The α and β WGDs identified in Arabidopsis [43] occurred in the Brasicales lineage, but neither are shared with papaya [44, 45]. The eucalyptus and poplar genomes have undergone an additional WGD event not shared with other species used in the analysis [13, 18]. The papilionoids underwent a WGD that is shared by soybean and medicago, but a more recent WGD event occurred in the soybean lineage (the milletioids) only [17, 46].^a^ The number of chestnut marker alignments to the other genome used in construction of predicted syntenic blocks. ^b^ The number of chestnut BES alignments to the other genome used in construction of a predicted syntenic blocks. ^c^ The percent of the chestnut physical map aligned to at least one location in the other genome, measured in consensus band units. ^d^ The percent of the chestnut physical map aligned to two or more locations in the other genome, measured in consensus band units. ^e^ The percent of the other genome aligned to at least one chestnut physical map contig, measured in bases. ^f^ The percent of the other genome aligned to two or more chestnut physical map contigs, measured in bases

The chestnut genome aligned best to the peach genome in overall percentage by a slight margin over grape (Fig. 1). Of the 1300 physical map contigs, 288 aligned to the peach genome. When a set of physical map contigs were sequentially anchored across a chestnut linkage group and also sequentially aligned to a peach chromosome location, they were placed into a putative syntenic block (dot plots illustrating blocks are provided in Additional file 1). A total of 93 such syntenic blocks were created based on 1,472 BES and 639 BES alignments between the genomes. When measured against the peach genome, 13 blocks spanned over 3 Mb, 19 blocks spanned 1 to 3 Mb and 61 blocks spanned less than 1 Mb. About 39 % of the chestnut physical map span was contained in syntenic blocks as measured in consensus band units. The blocks covered 35 % of the peach genome. Some genomic segments unique in one species mapped to two segments in the other genome. The chestnut physical map had 4 % double coverage of the peach, while the peach genome double covered 5 % of the chestnut genome.

**Fig. 2:**
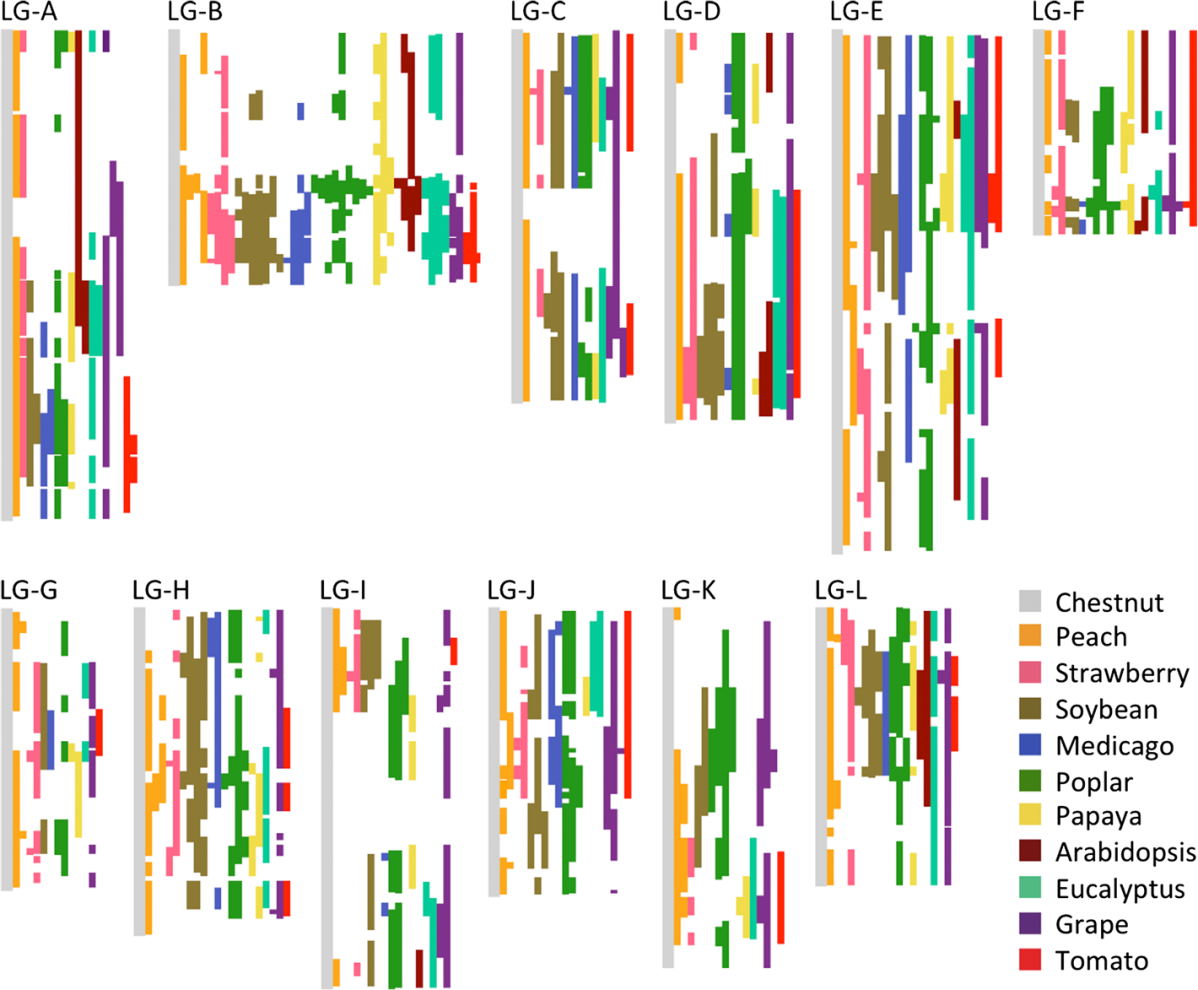
Location of predicted syntenic genome blocks. Conserved blocks determined by the Symap software from each chestnut linkage group to the ten aligned species are illustrated by colored blocks

Despite being phylogenetically distant to chestnut compared to peach and other species in the analysis, the same percent of the chestnut genome, 39 %, aligned to grape as to peach. The grape comparison also yielded the second highest number of aligned chestnut physical map contigs, 280. The genome organization of chestnut to poplar was also surprisingly conserved with 36 % of the chestnut genome aligning into 134 large syntenic blocks. The recent whole genome duplication in poplar is evident, with 21 % of the chestnut genome aligning to the poplar genome in two locations. The chestnut regions with synteny to two populus chromosomes often correspond to the regions of poplar known to have arisen from the WGD [13] (Additional file 2). This double coverage in poplar is 4 times more extensive than the 4 and 7 % double coverage that was detected with peach and grape, respectively. The syntenic blocks between poplar and chestnut were larger than the syntenic blocks found for peach, an expected outcome given that poplar has a larger genome. Of the syntenic blocks found between poplar and chestnut, 24 blocks were over 3 Mb in length, 40 blocks ranged in length from 1 Mb to 3 Mb, and 70 blocks were less than 1 Mb. Less conservation of synteny was observed with the other species studied, with less than 20 % of the chestnut genome aligning to the Medicago, papaya, Arabidopsis and tomato genomes (Fig. 2).

Some physical map contigs consistently aligned. Twelve physical map contigs aligned to all ten comparison species. Six of these 12 contigs corresponded to the QTL contigs, which were preferentially saturated with overgo markers and BES (discussed further below). The 12 contigs that aligned to all of the reference genomes studied were both significantly longer than the average physical map contig (1466 CBs versus 823 CBs) and contained more overgo markers (18 versus 3 average overgos/contig). Due to the overgo probes being predominantly EST-based, these contigs may represent gene rich regions and contain less repetitive sequence. It is possible that these regions retained conserved genomic structure on a macrosytentic scale across dicots.

### QTL sequences

The chestnut genetic linkage map was initially constructed by Kubisiak et al. [14] from an F_2_ family of an American x Chinese chestnut cross using RFLPs and RAPDs [14]. Inoculation and phenotyping of the plants were carried out to identify QTLs conferring resistance to blight. Three main regions in the genome associated with variation in phenotype were found, one each on linkage groups B, F and G. The same population was genotyped using SNPs from the updated high resolution genetic map, and the QTL analysis was recalculated with the new markers [8]. The locations of the three QTL regions were refined to LG-B from 40.9 to 50.4 cM, LG-F from 38.1 to 46.8 cM and LG-G from 35.7 to 39.5 cM. The three QTLs are referred to as *Cbr*1, *Cbr*2 and *Cbr*3, respectively.

Emphasis was placed on thoroughly and accurately defining the QTL regions of the genetic map within the physical map framework to enable targeted sequencing. Sequence data from this region could help American chestnut restoration efforts in several ways, including development of markers for marker assisted selection, using additional markers for fine mapping of the QTLs and identification of genes directly involved in blight resistance. To identify the physical map contigs within these three QTL regions, the ESTs containing the SNPs and SSRs that were genetically mapped in QTL regions were used for BAC library overgo probe design. For each selected physical map contig, a MTP of BACs was selected for pooling and sequencing. The pools of BACs from each of the three QTL regions were sequenced, yielding a total of 4.3 billion bases (Additional file 3) [SRA:SRR1646589-SRR1646592, SRR1646621-SRR1646628]. Pools C and D were analyzed together because they originate from contiguous genomic sequence spanning *Cbr*1. The *Cbr*1 sequence data assembled into 214 scaffolds spanning 6.77 Mb. The pool B sequences, representing *Cbr*2, assembled into 128 scaffolds totaling 4.12 MB. Data from pool A from *Cbr*3 was assembled into 2.99 Mb in 53 scaffolds (Table 1).

**Table 1:** Assembly of sequences spanning the three blight resistance QTL regions in the Chinese chestnut genome

QTL target	cbr1	cbr2	cbr3
Genetic map location	LGB (40.9-50.4 cM)	LGF (38.1-46.8 cM)	LGG (35.7-39.5 cM)
BAC Pool	CD	B	A
Scaffolds	214	128	53
avg length	31,657	32,151	56,410
N50	75,056	72,331	158,218
Total length	6,774,520	4,115,273	2,989,748

The ends of BACs from the QTL minimum tiling paths were forward and reverse end sequenced using Sanger technology. These sequences were compared to the next generation sequence data. The majority of the BAC ends were found in the sequence assemblies: 92 % for *cbr*1, 86 % for *cbr*2 and 92 % for *cbr*3. This affirmed that end sequence was produced from the majority of the BACs in each pool, and that the coverage across the minimum tiling paths were high.

The assembled genomic sequence scaffolds from the BAC pools were annotated for repeat elements and genes. Over 10 % of the sequence was identified as interspersed repeats, comprising 8.45 % retroelements and 1.26 % DNA transposons. LTR elements, common in many plant genomes, encompassed 7.1 % of the bases with slightly more Gypsy elements (4.06 %) identified than Copia elements (2.69 %). Over 9,000 microsatellite sequences were identified in the scaffolds, yielding an average of one microsatellite every 1,492 bases.

Across all three sequenced regions, 782 genes were annotated (432 in *cbr*1, 219 in *cbr*2, 131 in *cbr*3). Functional annotation of the genes showed a diversity of molecular functions and biological processes (Additional file 4). Fifteen of the genes were annotated with the “defense response” GO term (Table 2). These genes are of particular interest for further study of blight resistance.

**Table 2:** Genes in the QTL sequences annotated *in silico* with the gene ontology term “defense response”

Seq. name	Seq. description	Closest matching NCBI nr protein
cbr1_scaffold114-gene-0.3-mRNA-1	Transcription factor tga1	Transcription factor TGA1 (Vitis vinifera)
cbr1_scaffold134-gene-0.0-mRNA-1	cc-nbs-lrr resistance protein	Putative disease resistance protein RGA3 (Vitis vinifera)
cbr1_scaffold16-gene-0.12-mRNA-1	rna recognition motif-containing protein	PREDICTED: DAZ-associated protein 1-like (Vitis vinifera)
cbr1_scaffold17-gene-0.29-mRNA-1	Beta-hydroxyacyl-acp dehydratase	Predicted protein (Populus trichocarpa)
cbr1_scaffold28-gene-0.12-mRNA-1	Transcription factor tga1	TGA transcription factor 1 (Populus tremula x Populus alba)
cbr1_scaffold32-gene-0.28-mRNA-1	14-3-3-like protein gf14 lambda	Hypothetical protein ARALYDRAFT_496774 [Arabidopsis lyrata subsp. lyrata)
cbr1_scaffold4-gene-0.38-mRNA-1	Multicatalytic endopeptidase complex	Proteasome subunit alpha type-7 (Vitis vinifera)
cbr1_scaffold61-gene-0.11-mRNA-1	Disease resistance protein at4g27190-like	PREDICTED: disease resistance protein At4g27190-like (Vitis vinifera)
cbr2_scaffold29-gene-0.6-mRNA-1	cc-nbs-lrr resistance protein	cc-nbs-lrr resistance protein (Populus trichocarpa)
cbr2_scaffold34-gene-0.3-mRNA-1	Feronia receptor-like kinase	Serine/threonine-protein kinase PBS1, putative (Ricinus communis)
cbr2_scaffold3-gene-0.42-mRNA-1	Protein	PREDICTED: MLO protein homolog 1-like (Glycine max)
cbr2_scaffold5-gene-0.9-mRNA-1	Transferring glycosyl	Transferase, transferring glycosyl groups, putative (Ricinus communis)
cbr3_scaffold1-gene-1.1-mRNA-1	Histone-lysine n-methyltransferase ashh2-like	PREDICTED: uncharacterized protein LOC100245350 (Vitis vinifera)
cbr3_scaffold1-gene-1.19-mRNA-1	Set domain protein	PREDICTED: uncharacterized protein LOC100245350 (Vitis vinifera)
cbr3_scaffold28-gene-0.8-mRNA-1	Cysteine proteinase rd19a	Cysteine proteinase RD19a (Arabidopsis thaliana)

### QTL sequences and symap analysis

The sequence data obtained for the QTL provides an opportunity to assess microsynteny from the chestnut genome to reference plant genomes on a base pair level, and to ask how microsynteny results compare with the macrosynteny results from the physical map alignment analysis. Peach provided the best reference genome in this analysis, with the highest percent of chestnut bases aligned in total and the highest percent of chestnut bases aligned uniquely. This suggests fewer rearrangements between chestnut and peach than between chestnut and the other genomes studied. The known genome duplication events since last divergence between chestnut and poplar as well as between chestnut and soybean are evident by the percent of chestnut BESs aligning to multiple locations in those genomes.

**Fig. 3:**
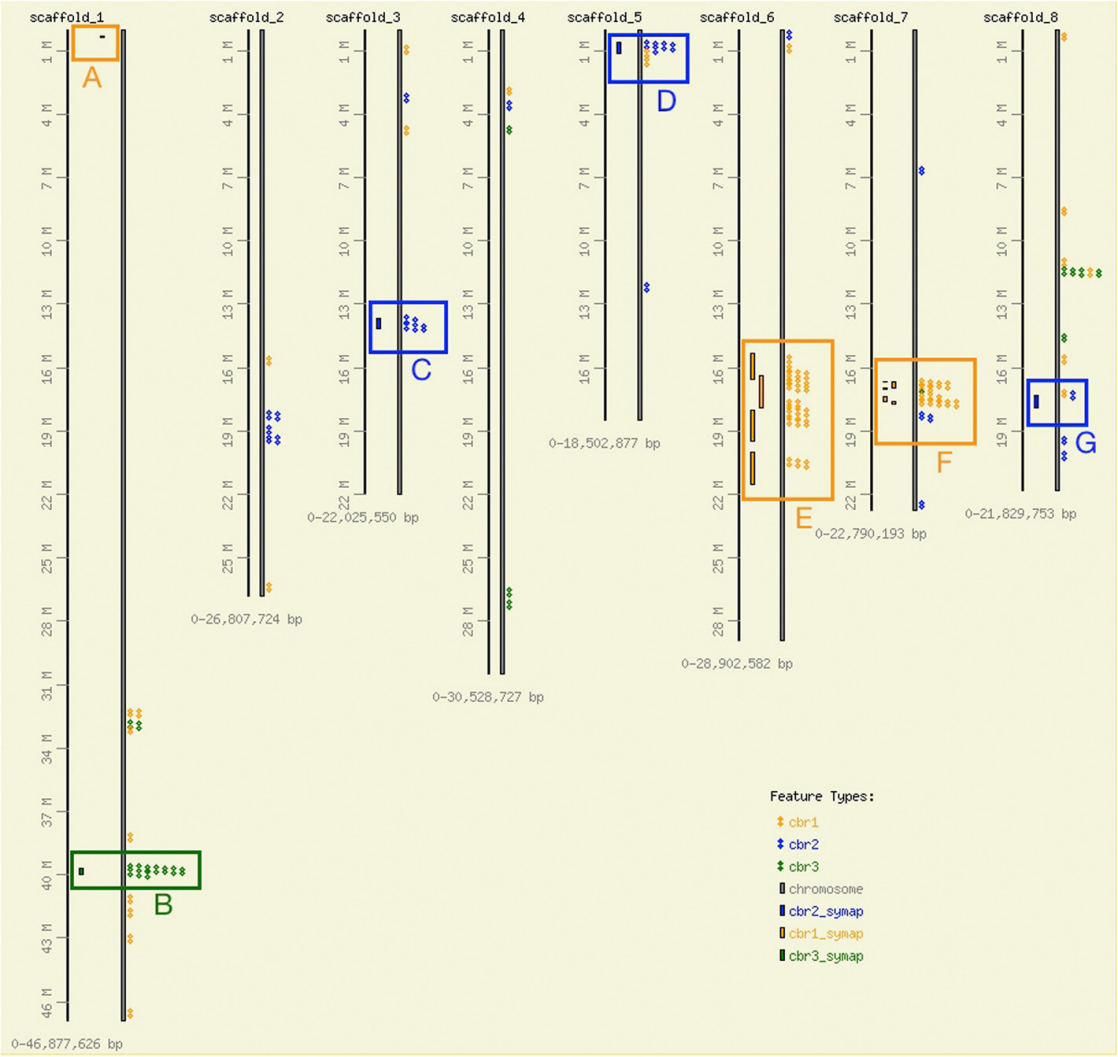
Alignments of the chestnut QTL regions to the peach genome. The chestnut QTLs are aligned to the peach genome in distinct clusters. Alignments found by the SynMap software using the sequence data are small blocks to the right of the chromosome. Alignments found by the Symap software using the integrated physical and genetic map are the colored blocks to the left of the chromosome. **a**. Symap prediction of synteny from physical map contig 11956 (cbr1) to peach scaffold_1 from 294 kb to 361 kb. This prediction is not supported by sequence evidence. **b**. Region of synteny identified by sequence similarity from cbr3 from 39.7 Mb to 40.0 Mb on peach scaffold_1. Symap predicted synteny for ctg7039 from 39.7 Mb to 40.0 Mb. **c**. Region of synteny identified by sequence similarity from cbr2 from 13.7 Mb to 14.1 Mb on peach scaffold_3. Symap predicted synteny for ctg403 from 13.7 Mb to 14.1 Mb. **d**. Region of synteny identified by sequence similarity from cbr2 from .7 Mb to 1.0 Mb on peach scaffold_5. Symap predicted synteny for ctg403 from .6 Mb to 1.2 Mb. **e**. Region of synteny identified by sequence similarity from cbr1 from 15.6 Mb to 18.7 Mb on peach scaffold_6. Symap predicted synteny in four sections: ctg11956 (15.4 Mb to 16.5 Mb and 20.0 Mb to 21.5 Mb), ctg9166 (16.4 Mb to 17.0 Mb), ctg4269 (18.0 Mb to 19.5 Mb). **f**. Region of synteny identified by sequence similarity from cbr1 from 16.7 Mb to 17.7 Mb on peach scaffold_7. Symap predicted synteny in five sections: ctg11956 (16.685 Mb to 16.692 Mb and 17.376 Mb to 17.592 Mb), ctg3279 (16.699 Mb to 16.945 Mb), ctg4269 (16.959 Mb to 17.007 Mb), ctg9166 (17.611 Mb to 17.718 Mb). **g**. Region of synteny identified by sequence similarity from cbr2 from 17.30 Mb to 17.31 Mb on peach scaffold_8. Symap predicted synteny for ctg403 from 17.3 to 17.9 Mb

By visualizing the locations of the syntenic alignments from chestnut to peach, larger areas of contiguous homology emerged where the chestnut scaffolds map within close proximity on a peach scaffold. Fig. 3 displays the regions of the peach genome with putative homology to the QTL regions, via either sequence homology (diamonds) or a Symap prediction (bars). The predicted syntenic regions from the Symap analysis were supported by the sequence alignment in all cases except one (Fig. 3a) where Symap identified a small region on peach chromosome 1 that the sequence mapping did not.

Alignments of chestnut *cbr*1 clustered in two main regions of the peach genome - scaffold 6 from 15.6 to 18.7 Mb and scaffold 7 from 16.7 to 17.7 Mb. The homology to peach scaffold 6 (from 16.5 Mb to 18.6 Mb) was previously identified in the recent genetic linkage map paper [8]. The two previously reported regions of alignment to peach scaffold 7 (16.7-16.8 Mb and 17.6-17.7 Mb) now appear to be part of one single syntenic block on peach scaffold 7. Chestnut sequence scaffolds containing 4.8 Mb were mapped, representing 70 % of the cbr1 bases. Of the annotated *cbr*1 genes, 52.1 % match genes most closely in these two peach genome regions.

**Fig. 4:**
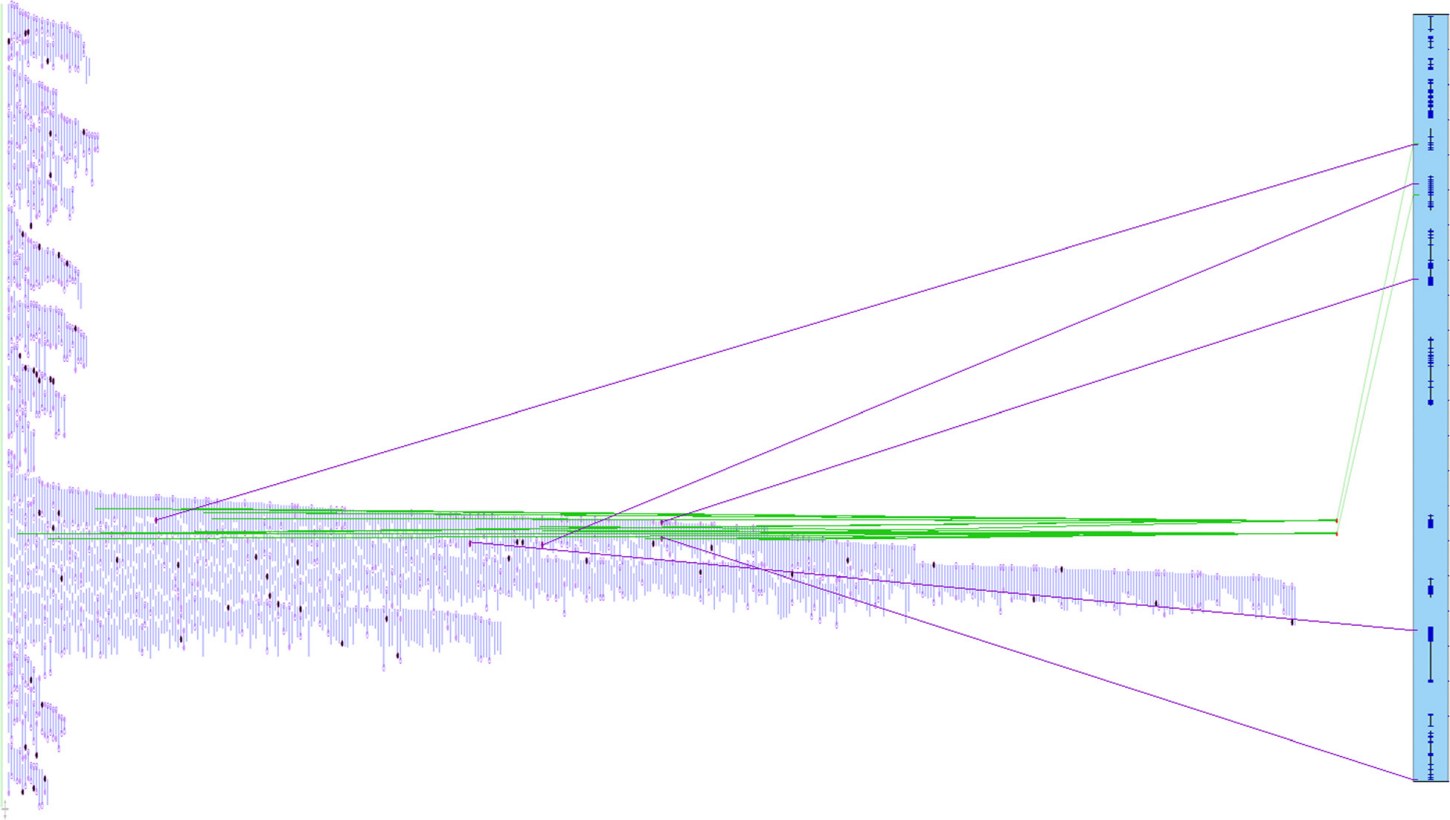
Topology of physical map ctg11956 and matches to peach scaffold 1. The physical map assembly of the ctg11956 is visualized as short, parallel blue lines representing individual BAC clones. The peach chromosomal segment, scaffold 1 from 294 kb to 361 kb, is represented as blue bar on the right with genes as small dark blue boxes. The alignment of the physical map contig to peach is based on sequence matches of two overgo probes: CCall_contig366_v2 (source of CmSNP00620) and CCall_contig3045_v2 (source of CmSNP00614), represented as green lines from the BACs with positive overgo probe hits to the sequence location on the peach segment. Additional supporting evidence is provided by aligned BAC end sequences (purple lines from individual BACs to peach)

The Symap results from the 4 physical map contigs covering *cbr*1 also map primarily to scaffold 6 and 7 in peach. However, for scaffold 6, Symap predicted alignments from 15.4 Mb to 21.5 Mb, significantly larger than the sequence alignments (Fig. 3e). The Symap-identified region from scaffold 7, 16.7 Mb to 17.7 Mb is nearly identical to that predicted by the sequence alignments (Fig. 3f). In one instance Symap predicted a putative syntenic region between *cbr*1 and peach scaffold 1 where there is no sequence alignment confirmation (Fig. 3a). The Symap alignment, from 294 kb to 361 kb on peach scaffold_1, is based on sequence alignments to two overgo probes: CCall_contig366_v2 (source of CmSNP00620) and CCall_contig3045_v2 (source of CmSNP00614). Neither EST sequence from these two probes has sequence similarity to the raw or assembled sequences from the BAC pools. It appears that this region was missed in sequencing. An examination of the topology of the physical map contig shows that these two probes matched a set of BACs aligned with many hundreds of other BACs. This type of BAC assembly is generally due to repetitive elements and results in a contig where hundreds or thousands of BACs are incorrectly “collapsed” into a small consensus region (Fig. 4). Thus, by selecting only a minimal tiling path across the contig, the resulting sequencing was likely not representative of the entire genomic sequence in that region. It is likely that the unique genomic regions containing the missing markers is flanking or between the repetitive elements that caused the incorrect assembly, and by being buried in the deep stack of incorrectly assembled BACs, they were not represented in the sequencing pool.

The previously identified syntenic region for *cbr*2 was against scaffold 2 from 17.9 to 20.0 Mb in peach. This block was confirmed in the current analysis, but two other regions also emerged as putative homologous areas. The three main syntenic regions found on peach for *cbr*2 in this analysis are scaffold 2 from 18.2 to 19.4 Mb, scaffold 3 from 13.7 to 14.1 Mb, and scaffold 5 from .7 to 1.0 Mb. The scaffold 2 syntenic region was not predicted by Symap, but the other two were predicted with very similar borders: scaffold 3 from 13.7 to 14.1 Mb (Fig. 3c) and scaffold 5 from .6 to 1.2 Mb (Fig. 3d). Symap did predict a region of synteny for *cbr*2 on peach scaffold 8 from 17.3 to 17.9 Mb that was not entirely supported by sequence evidence (Fig. 3g). One sequence scaffold, *cbr*2_scaffold22, aligned near position 17.3 Mb, but no other sequence agreed with the Symap prediction. The scaffolds placed in the three major syntenic regions in peach represent 56.9 % of the *cbr*2 assembly and 59.4 % of the *cbr*2 genes had a best match in peach within those three regions.

*Cbr*3 had the most clearly placed homologous region in peach. The sequence alignments and the Symap alignment agreed that it aligns to peach scaffold 1 from 39.7 to 40.0 Mb (Fig. 3b). Of all the bases in *cbr*3, 66.6 % were in scaffolds aligning to this region and 42.0 % of *cbr*3 genes aligned best to that region of peach.

Overall, Symap was effective at identifying most of the regions predicted by sequence alignments. It predicted syntenies in 5 of 6 regions, although of significantly increased size in one instance. It also predicted 1 region of synteny missed from the partial sequence coverage of the QTL regions. Had the comparative Symap results not been available for analyses, the absence of this gene information in the QTL sequences may not have been identified. Syntenic analysis provides both an avenue for transferring information across genomes and for validating new information.

Two main QTLs in peach have been identified for resistance to powdery mildew (*Sphaerotheca pannosa* var. *persicae*), located on scaffold 6 from 16.0 to 22.94 Mb and scaffold 8 from 11.48 to 17.21 Mb [15]. As discussed in [8], two of the chestnut QTLs overlap these two QTLs from peach. The *cbr*1 QTL from chestnut aligns to a significant portion of the same region of scaffold 6 (15.6 to 18.7 Mb). *Cbr*2 aligns to the end of the peach powdery mildew QTL on chromosome 8.

## Discussion

Chinese chestnut has emerged as a model species for the family Fagaceae. Naturally resistant to the chestnut blight (*Cryphonectria parasitica*), it has become particularly important for restoration of American chestnut (*Castanea dentata*). A backcross breeding strategy utilizing Chinese chestnut as the source of natural resistance has been ongoing since the 1960s. Development of molecular tools to reduce time and space requirements for breeding are vital to quickly advancing American chestnut restoration efforts and for advancing our genetic understanding of resistance to other chestnut pathogens such as *Phytophthora cinnamomi*. Here we report on new Chinese chestnut genomic data sets including an improved integrated genetic/physical map and genomic sequence across QTL regions. These resources allow the chestnut genome to be compared on both a micro- and macro-synteny scale to other genomic model plant species.

The integrated physical and genetic map enabled a targeted sequencing approach for the QTL regions conferring resistance to chestnut blight. Utilizing overgo hybridization probes corresponding to genetic map markers, six physical map contigs from the QTL regions were selected. From these a minimum tiling path of BACs was pooled and sequenced. The resulting assembled genomic sequence scaffolds provide a list of candidate genes to pursue for further understanding of the molecular underpinnings of blight resistance. These candidate genes have been tentatively prioritized through functional profiling and assignment of Gene Ontology terms. Downstream analysis to explore the role of these genes is ongoing. Comparative genomic approaches as well as sequencing of the corresponding homologous sequences in American chestnut may help to further elucidate the source of resistance to blight in Chinese chestnut.

Identifying and sequencing BACs across a region of interest is a cost-effective strategy for obtaining information on the genes and genomic organization in these important regions without undertaking an expensive and complex whole genome sequencing effort. American chestnut is plagued by biotic challenges beyond chestnut blight. The oomycete *Phytophthora cinnamomi* causes root rot with a highly mortality rate and is widespread through the native range of American chestnut, presenting a significant barrier to any American chestnut reintroduction [2]. Chinese chestnut can serve as a source of resistance to this pathogen as well as to chestnut blight [16]. If QTL regions of resistance are found using a genetic mapping approach, the same BAC pooling and sequencing approaches could be used once again. The sequenced regions for blight resistance as well as any future desirable QTL have an additional utility beyond candidate gene discovery; they will enable discovery of markers for fine mapping and marker assisted selection. These markers will be essential for large scale backcross breeding efforts to produce a tree of primarily American chestnut origin but with a combination of Chinese chestnut chromosomal segments that confer necessary biotic resistance loci.

The genomic resources for Chinese chestnut also facilitate a survey of macrosynteny retained between chestnut and other species with sequenced genomes. Analysis of peach, strawberry, soybean, Medicago, poplar, papaya, Arabidopsis, Eucalyptus, grape and tomato found putative syntenic blocks in all species. Peach, the phylogenetically-closest hardwood tree from this list, showed the highest amount of conservation with chestnut. The percent of duplicated blocks between the two genomes indicates that no whole genome duplication events (WGDs) have taken place since the divergence of chestnut and peach from their last common ancestor. Poplar and soybean have both undergone recent whole genome duplications, and these events can be detected by the significantly larger amount of chestnut genomic sequence that can be aligned to two separate locations. In poplar, the WGD occurred 60 to 65 Mya [13], and over half of the chestnut syntenic blocks can be detected in two locations. For soybean, WGDs have occurred twice, at 13 and 59 Mya [17]. The majority of chestnut blocks were detected in two locations, but interestingly, they were not found in three or four locations as might be expected from the occurrence of two WGDs. This is likely due to the ongoing diploidization of the soybean genome, where the majority of homologous genes (61.4 %) were found to exist in blocks involving only two locations while a much smaller percentage were found in blocks in three or four locations (5.63 and 21.53 %, respectively) [17]. The maintenance of multiple regions of synteny with chestnut is not detectable in the other genomes that have undergone a WGD. Very few chestnut regions are aligned to multiple locations in the eucalyptus genome, which has evidence of a WGD 110 million years ago (Mya) [18], or to the tomato genome, which underwent a whole genome triplication around 71Mya [19]. The lack of evidence of these events may be due to the large phylogenetic distance between chestnut and these species or the rate at which these genomes have undergone diploidization.

While the detection of WGDs does appear to depend on phylogenetic distance, the overall number and length of conserved genomic blocks between species did not always correspond to the phylogenetic distance. For eight species, greater than 87 % of the genomic sequence anchored to pseudo-chromosomes and genomic sequence comprised of less than 7 % N’s (unknown nucleotides). The quality of genomic alignments is likely to be comparable across these eight. The Medicago genome is slightly more fragmented with 71 % of the genomic sequence in eight pseudo-chromosomes and 12 % Ns. The papaya sequence is highly fragmented, with 29 % Ns and no pseudochromosomes. Interestingly, the number of putative syntenic blocks between chestnut and papaya was higher than the blocks found against eucalyptus, another Malvidae with a more contiguous genome reference sequence.

The two best conserved genomes with 39 % of chestnut mapping to the other genome in syntenic blocks were peach and grape. Although the results with the relatively closely-related peach clearly represent the expected close genomic relationship with Chestnut, grape showed greater extent of genome structure preservation than would fit our expectations based on phylogeny. No WGDs have occurred in either chestnut or grape since their last common ancestor. The grape genome is known to be structurally conserved with very few rearrangements since the paleo-hexaploid ancester of all rosids [20]. The slow rate of structural rearrangements in grape that has maintained an ancestral state of organization is a possible reason for the extensive structural homology. However, papaya and strawberry also have not experienced WGDs since divergence from grape and the same level of conservation is not present. This may indicate that chestnut has a slower rate of structural reorganization over time, or another possible hypothesis is that grape is misplaced phylogenetically. In this last regard, it is interesting to note that grape (Vitaceae) was formerly botanically placed by Cronquist near the family Rhamnaceae in order Rhamnales [21], much closer to peach and chestnut than the other sequenced genomes in our study. Molecular studies led to its placement as an outgroup to the rosid I and II clades but this placement did not have strong support [22, 23]. This former phylogenetic position would support our findings concerning the degree of genome preservation among the study species.

## Conclusions

If in fact grape is not misplaced phylogenetically, then it is interesting to note a trend toward woody plant species maintaining a higher level of structural synteny with each other than with herbaceous species in the same family. In this regard, poplar is an additional example. Poplar has maintained a greater degree of genome similarity to chestnut than strawberry, soybean and Medicago, which are all closer to chestnut phylogenetically. This could possibly suggest that woody plant species evolution is somehow more constrained, or much slower, than the herbaceous species within the same family. However, for strawberry in particular, the lack of syntenic blocks could be due to the fact that the strawberry genome assembly is slightly more fragmented than the peach, with 7 % of the sequence comprised of unknown nucleotide gaps versus 1 % for peach. For the species at a further phylogenetic distance, chestnut has double the quantity of conserved genomic blocks with papaya and eucalyptus versus Arabidopsis. However, the *Arabidopsis thaliana* genome may not be representative of other herbaceous species, as it has undergone recent (<10Mya) large-scale rearrangements and DNA loss [20, 24]. Additional whole genome references from other woody and herbaceous angiosperm species is needed to conduct future comparative work and draw conclusions regarding the relationship of genome preservation to plant growth and habit. A contiguous whole genome sequence from chestnut and other members of the Fagaceae (e.g. oak) would also help to resolve rosid structural synteny on a finer scale.

## Methods

### Alignments of integrated physical and genetic map to reference genomes

Overgo probes were hybridized to the BAC clones and anchored to the physical map using the 3-dimensional pooling procedure described in [9]. Clones selected for BAC end sequencing were re-arrayed and cultured into 96-well plates. DNA was prepared with the ABI Dye Terminator chemistry and universal T7 and Sp6 primers. The results were resolved on an ABI 3730XL sequencer. A pipeline instituted for quality control performed base calling, filtering and vector/low-quality trimming with Phred, cross_match [25] and lucy [26]. The trimmed sequences with greater than 5 % uncalled bases (N’s) or less than 100 total bases were discarded. To identify conserved regions, the chestnut physical map, BES sequences, and overgo probe sequences were input to the Symap software [27] and aligned to reference plant genomes at default parameter settings. The genomes for peach (*Prunus persica*, version 139), strawberry (*Fragaria vesca*, version 226), soybean (*Glycine max*, version 189), Medicago (*Medicago truncatula*, version 198), poplar (*Populus trichocarpa*, version 210), papaya (*Carica papaya,* version 113), Arabidopsis (*Arabidopsis thaliana*, version 167), Eucalyptus (*Eucalyptus grandis*, version 201), grape (*Vitis vinifera*) and tomato (*Solanum lycopersicum*) were downloaded from Phytozome [28].

### QTL sequences

*Cbr*1 includes 20 genetic map markers, and 16 have successful corresponding overgo probes on the physical map. All 16 hybridize to one or more of the following four physical map contigs: ctg3279, ctg4269, ctg9166, ctg11956. A sequencing pool of 50 minimum tiling path (MTP) BACs was constructed representing ctg4269 and ctg9166 (pool C). A second sequencing pool of 49 MTP BACs from ctg11956 and 3279 was created (pool D).

For *Cbr*2, seven of eight genetic map markers have a successful overgo probe. All of the probes except one corresponded to physical map ctg403. A MTP of 51 BACs was picked from this contig and pooled for sequencing (pool B).

For *Cbr3*, alleles conferring resistance inherited from Chinese chestnut and LOD ±1.0 support intervals were localized to the region within 35.7–39.5 cM on LG-E of the Chinese chestnut consensus genetic map [8]. In total 26 genetic markers mapped into this interval were used for developing overgo probes for hybridizations. Of these, 18 probes were successfully hybridized. In total 9 physical contigs were pulled out for further consideration. The most associated markers CmSNP00127 and CmSNP01226 mapped to 37.4 cM on the consensus map were localized in physical map contig 7039. Forty BACs spanning contig 7039 were selected and pooled for sequencing (pool A). The genetic map markers in the QTL regions, their originating EST sequence names, and their physical map contig anchor points are in Additional file 5.

The minimum tiling path of each of the 6 physical map contigs was selected with FPC software [29]. After being picked, the clones were fingerprinted and compared to the original clone fingerprints used during map construction. The fingerprinting procedure is described in [9]. Four of the clones were found to be contaminated or had different fingerprints and were discarded. Six new clones that spanned the same areas of the physical map were selected to replace those four. Four total pools of DNA were created for sequencing. BAC addresses included in each pool can be found in Additional file 6.

Each of the four pools was prepared as a single end library and sequenced with ½ slide of 454 single end pyrosequencing on a Roche 454 GS FLX. Reads were screened for *E. coli* and the BAC vector pIndigoBAC5 using cross_match [25]. Additional sequencing was performed on a MiSeq instrument (Illumina); paired end libraries (2x250) were prepared from each DNA pool and run twice. The reads were trimmed with Trimmomatic [30]. The 454 data and the first run from the MiSeq were assembled with gsAssembler (Roche) using an overlap length of 30 and heterozygote mode. Using the second MiSeq data set, the contigs from the assembler were placed in scaffolds with the software SSPACE [31].

The resulting genomic sequence scaffolds were annotated for repeats and genes with the Maker pipeline [32]. Additional annotation was performed with Augustus [33], PASA [34], Gmap [35], and RepeatMasker [36] using RepBase [37]. Annotations from all software packages are available to be viewed on the hardwood genomics website [38] as tracks in Jbrowse [39]. The final gene calls from Maker are used as the final gene annotations for analysis; Blast2GO [40] was used to functionally annotate these genes.

Using CoGe’s SynMap tool [41], the QTL sequences were aligned to ten reference genome species. The ten genomes downloaded from Phytozome were: peach (*Prunus persica*), strawberry (*Fragaria vesca*), soybean (*Glycine max*), Medicago (*Medicago truncatula*), poplar (*Populus trichocarpa*), papaya (*Carica papaya*), Arabidopsis (*Arabidopsis thaliana*), Eucalyptus (*Eucalyptus grandis*), grape (*Vitis vinifera*) and tomato (*Solanum lycopersicum*). The DAGChainer output from SynMap was filtered to retain only the alignments scoring over 300.

## Availability of supporting data

The data sets supporting the results of this article are available in the GenBank repository, (accessions HN270092-HN275251 and JY172573-18703 at http://www.ncbi.nlm.nih.gov/genbank/) and in NCBI’s short reach archive (accessions SRR1646589-SRR1646592 and SRR1646621-SRR1646628 at http://www.ncbi.nlm.nih.gov/sra).

## Additional files

Additional file 1:
**Symap-generated dot plots for chestnut physical and genetic map against plant reference genomes.** (DOCX 297 kb) Additional file 2:
**Dot plots comparing each chestnut LG to the set of** ***Populus trichocarpa*** **chromosomes with highlightnig of areas of “double coverage” that correspond to the** ***Populus*** **WGD.** (DOCX 808 kb) Additional file 3:
**Illumina MiSeq and 454 sequencing output statistics.** (XLSX 42 kb) Additional file 4:
**The genes found in the QTL-targeted sequence pools and their predicted functions.** (XLSX 70 kb) Additional file 5:
**Genetic map markers defining the QTL region and corresponding physical map contigs.** (XLSX 40 kb) Additional file 6:
**The addresses of the BAC clones selected for sequencing.** (XLSX 58 kb)
