# Supplementary material for: Substantial genome synteny preservation among woody angiosperm species: comparative genomics of Chinese chestnut (Castanea mollissima) and plant reference genomes
Source: BMC Genomics. 2015 Oct 5;16:744. doi: 10.1186/s12864-015-1942-1 (PMC4595192; doi:10.1186/s12864-015-1942-1)

Symap generated dot plots for chestnut physical and genetic map against the peach genome


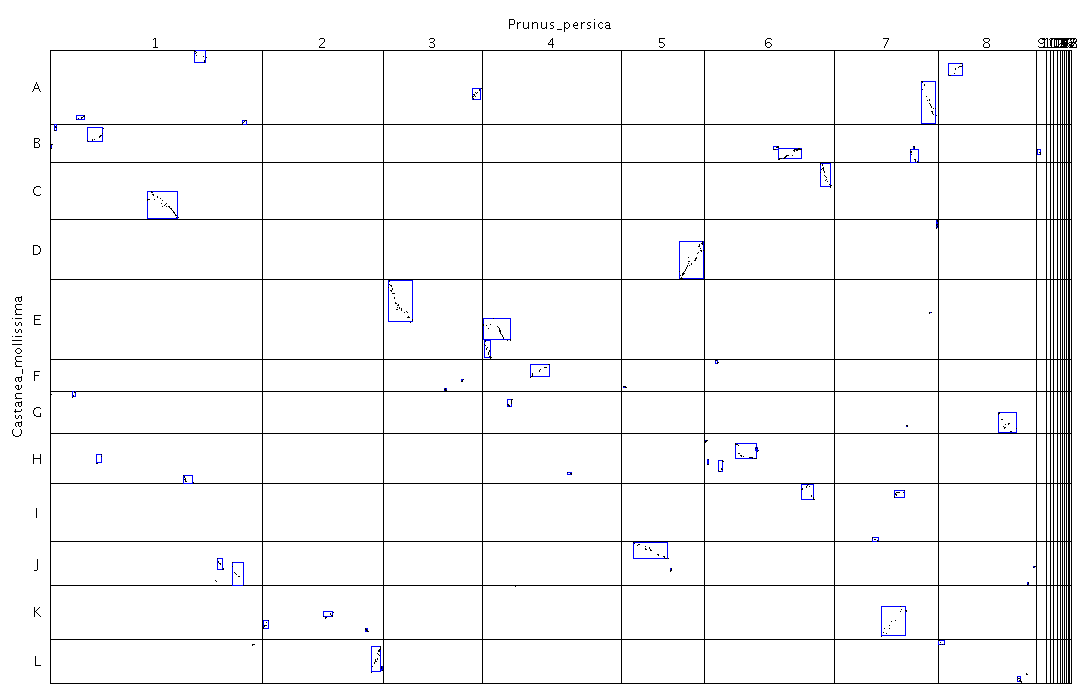


Symap generated dot plot for chestnut physical and genetic map against the strawberry genome


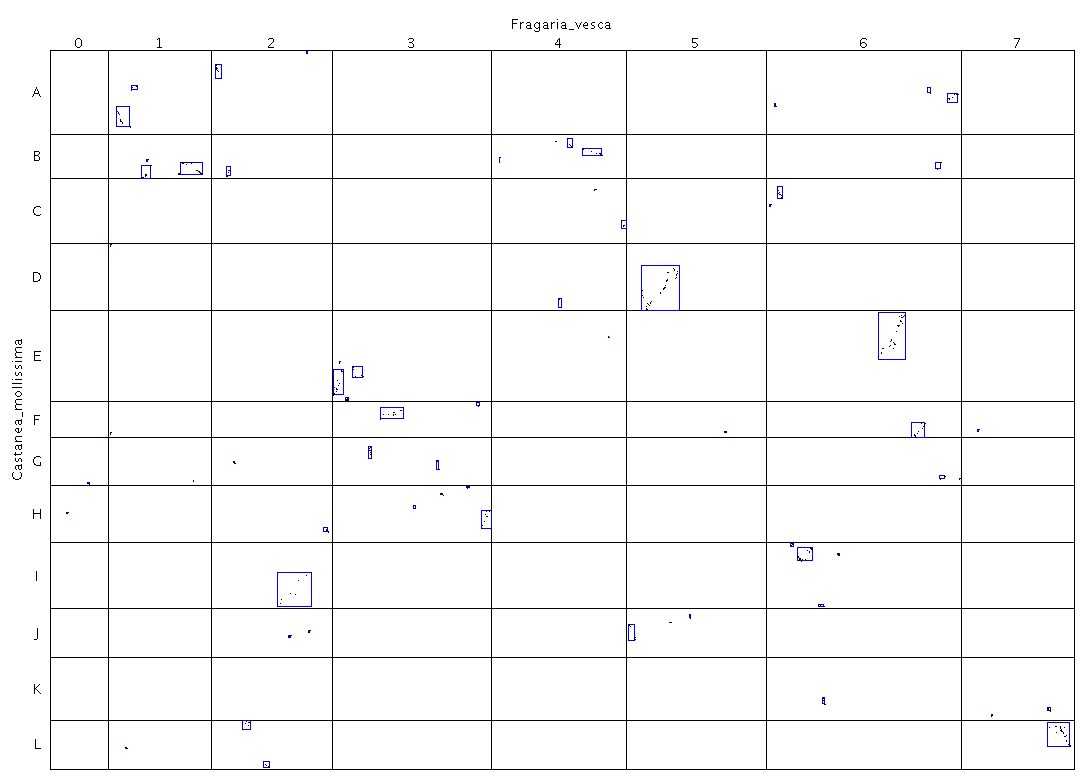


Symap generated dot plot for chestnut physical and genetic map against the soybean genome
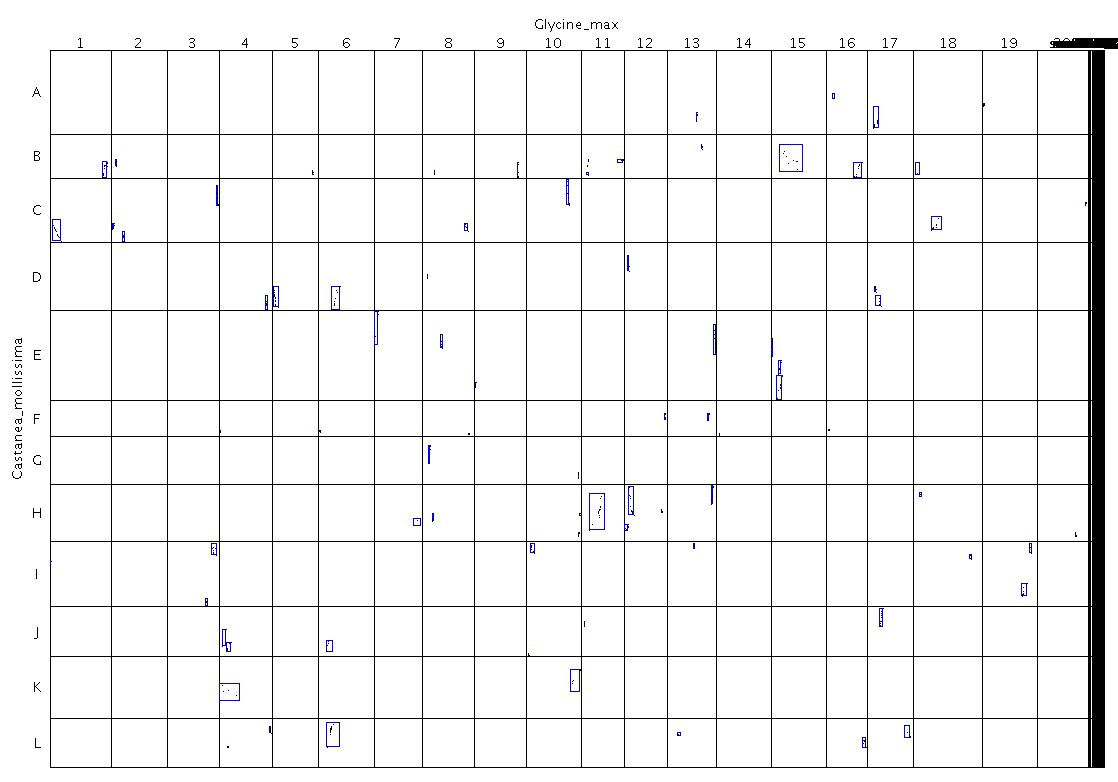


Symap generated dot plot for chestnut physical and genetic map against the medicago genome


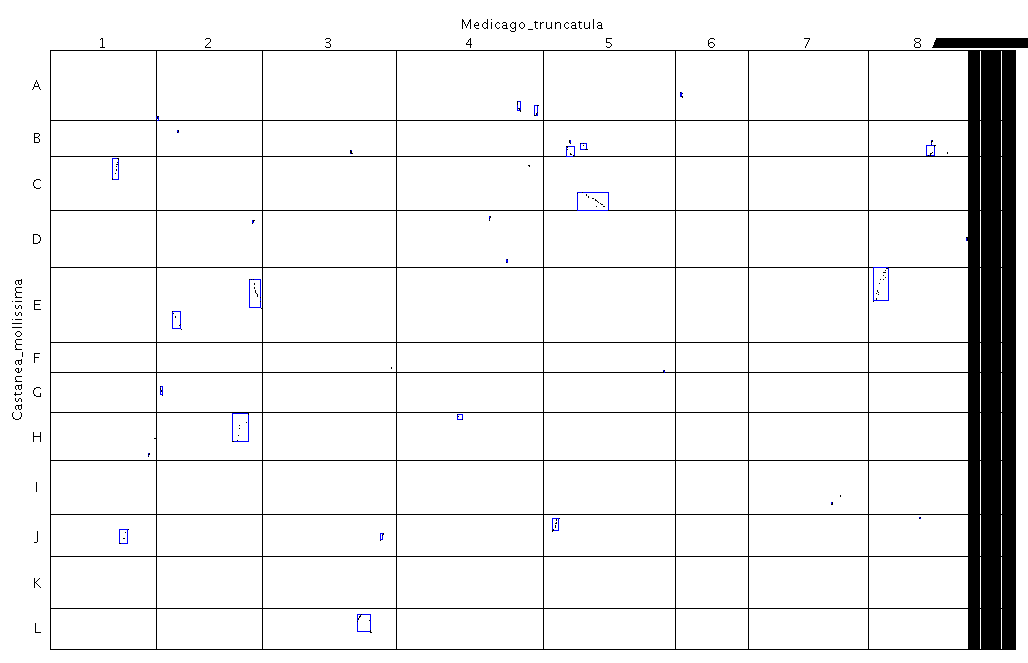


Symap generated dot plot for chestnut physical and genetic map against the poplar genome


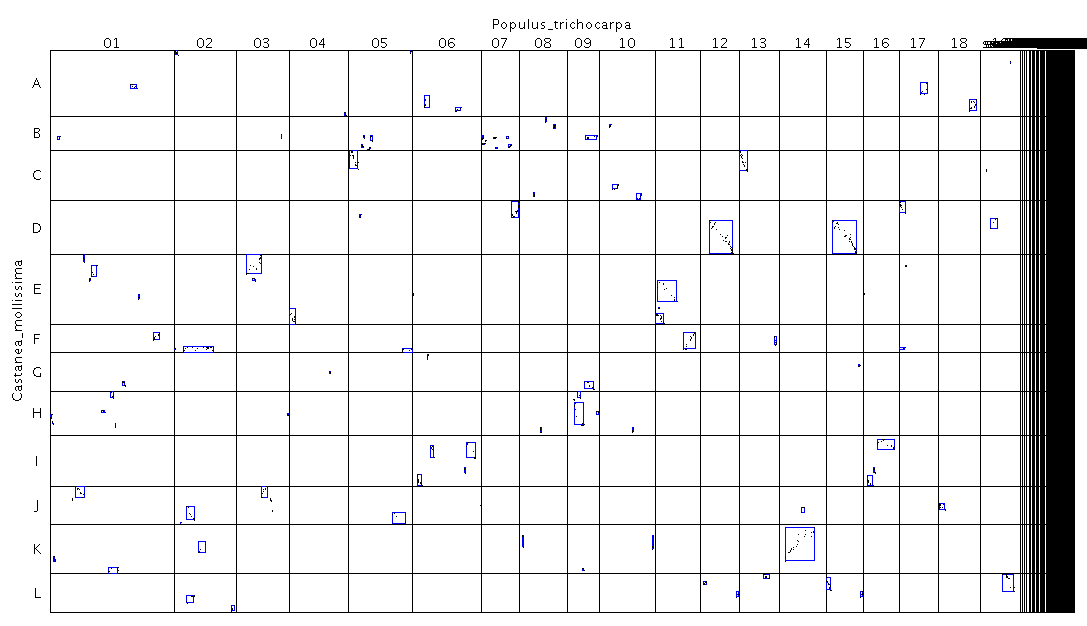


Symap generated dot plot for chestnut physical and genetic map against the papaya genome


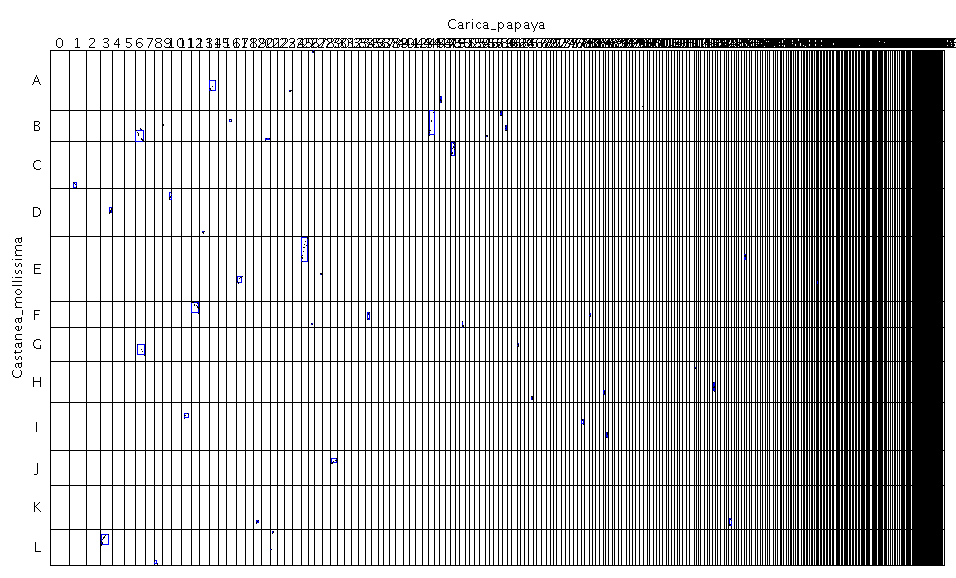


Symap generated dot plot for chestnut physical and genetic map against the Arabidopsis genome


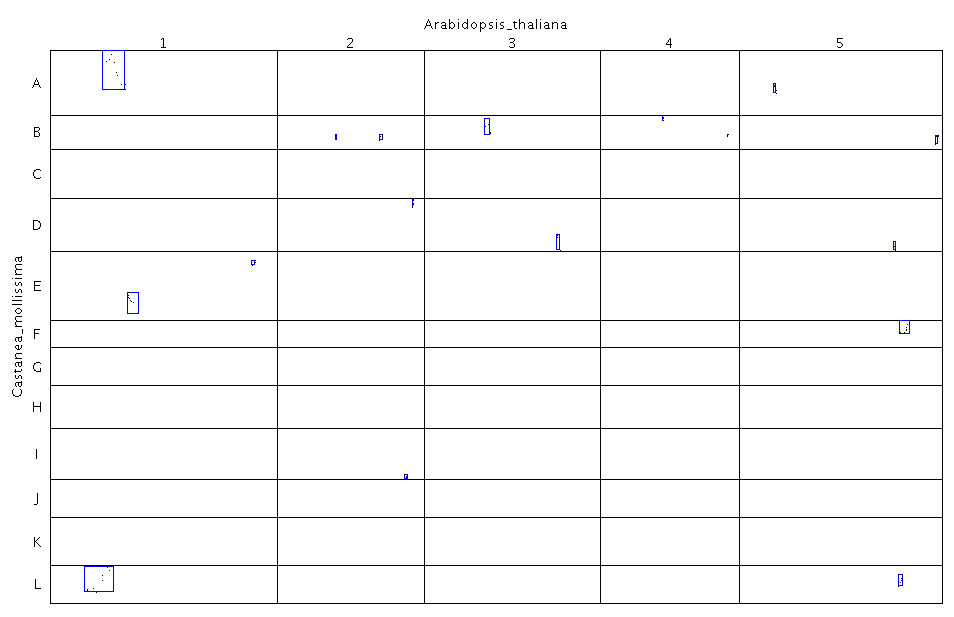


Symap generated dot plot for chestnut physical and genetic map against the eucalyptus genome


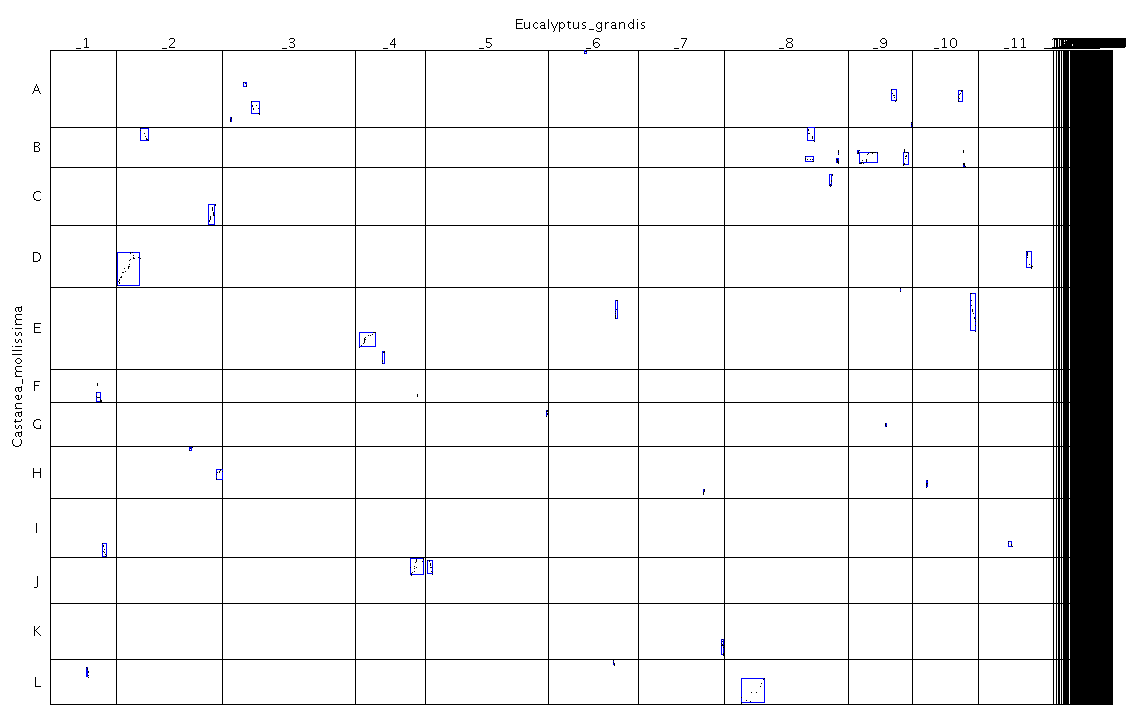


Symap generated dot plot for chestnut physical and genetic map against the grape genome


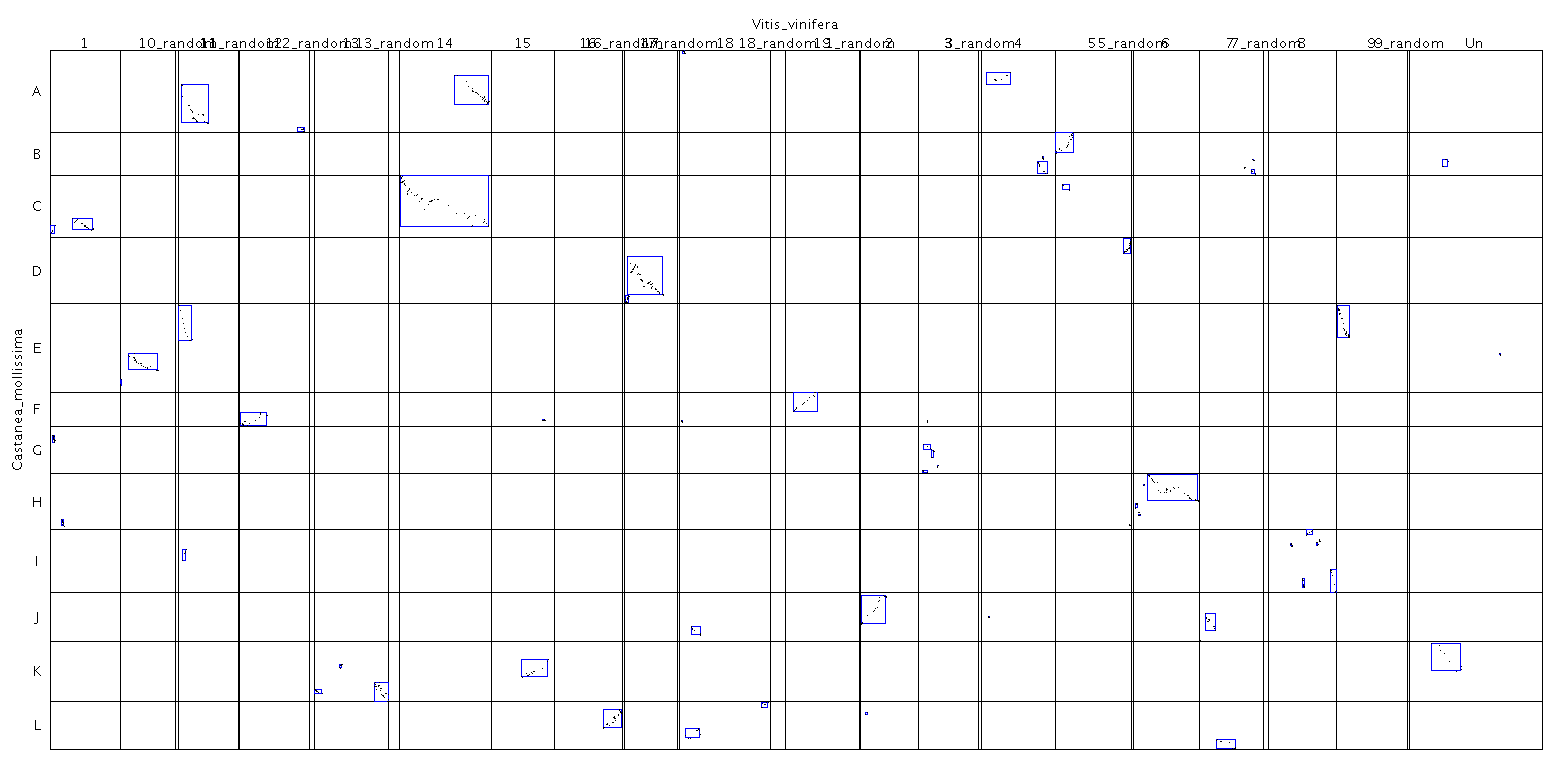


Symap generated dot plot for chestnut physical and genetic map against the tomato genome


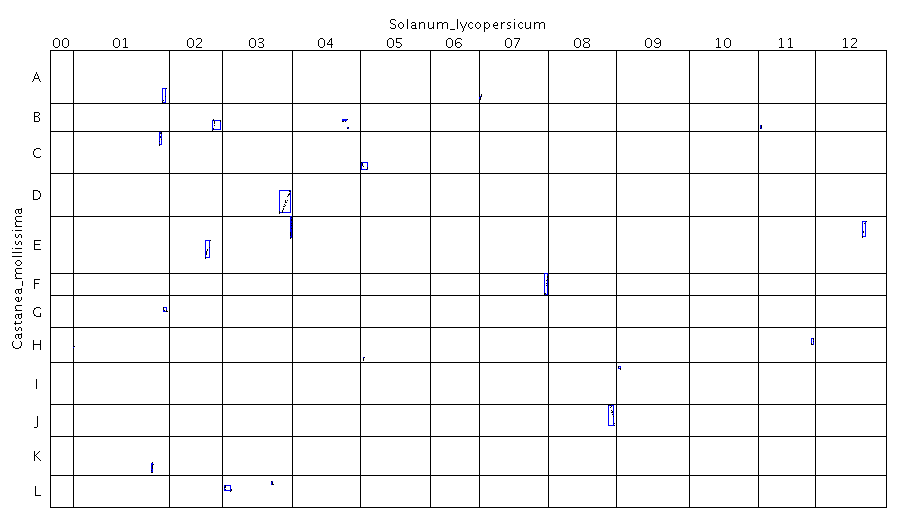

Supplement: Additional file 1: — Symap-generated dot plots for chestnut physical and genetic map against plant reference genomes. (DOCX 297 kb) [file 12864_2015_1942_MOESM1_ESM.docx]
