# Supplementary material for: Substantial genome synteny preservation among woody angiosperm species: comparative genomics of Chinese chestnut (Castanea mollissima) and plant reference genomes
Source: BMC Genomics. 2015 Oct 5;16:744. doi: 10.1186/s12864-015-1942-1 (PMC4595192; doi:10.1186/s12864-015-1942-1)

Dot plots comparing each chestnut LG to the set of *Populus trichocarpa* chromosomes. Blocks of putative synteny have been outlined in blue. (If no sytenic block was found for a populus chromosome, that chromosome was excluded to save space). In many cases, the same syntenic block for chestnut corresponds to two regions in populus. This is referred to as “double coverage” in the manuscript, and these regions often correspond to chromosomes known to share homology within the populus genome due to the most recent whole genome duplication in the Salicaceae.

Here, we have added colored blocks to indicate the double coverage regions and correlated those regions to the WGD map produced by Tuskan et al., 2006. Not all regions make sense in the scope of the WGD; see for example LGC and LGK. Additional resolution from a whole genome sequence of chestnut will be needed to determine exactly where the breakpoints of synteny are and where incorrect homology may have been identified. At that point, a map of chestnut chromosomes as they correspond to populus may be produced.

Chestnut Linkage group A.


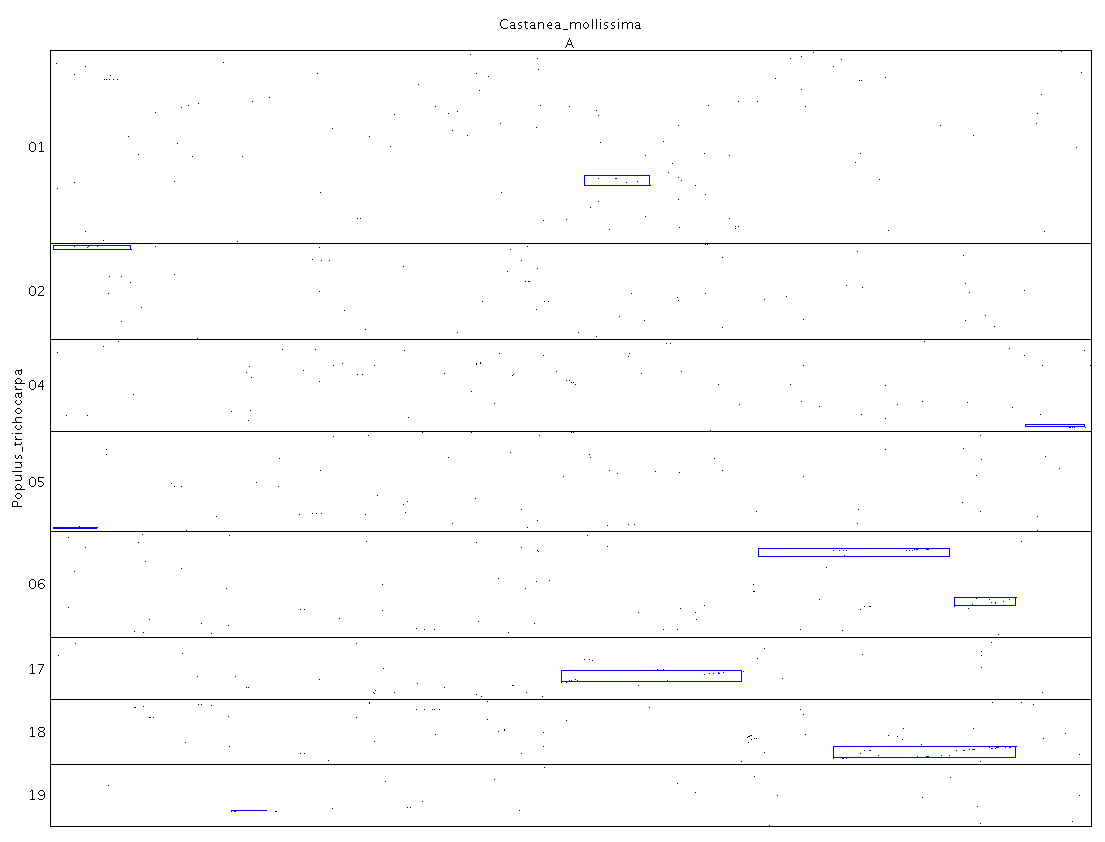

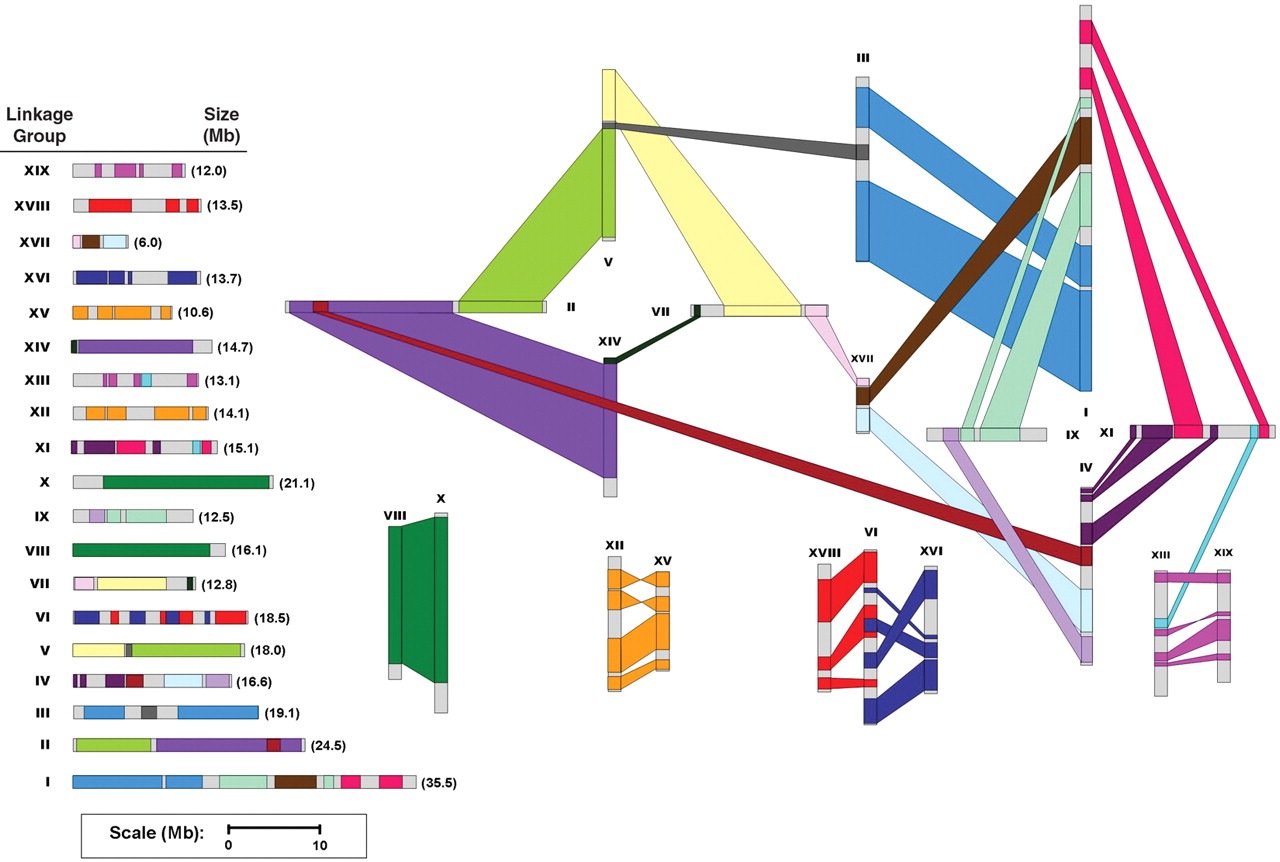


Chestnut Linkage group B.


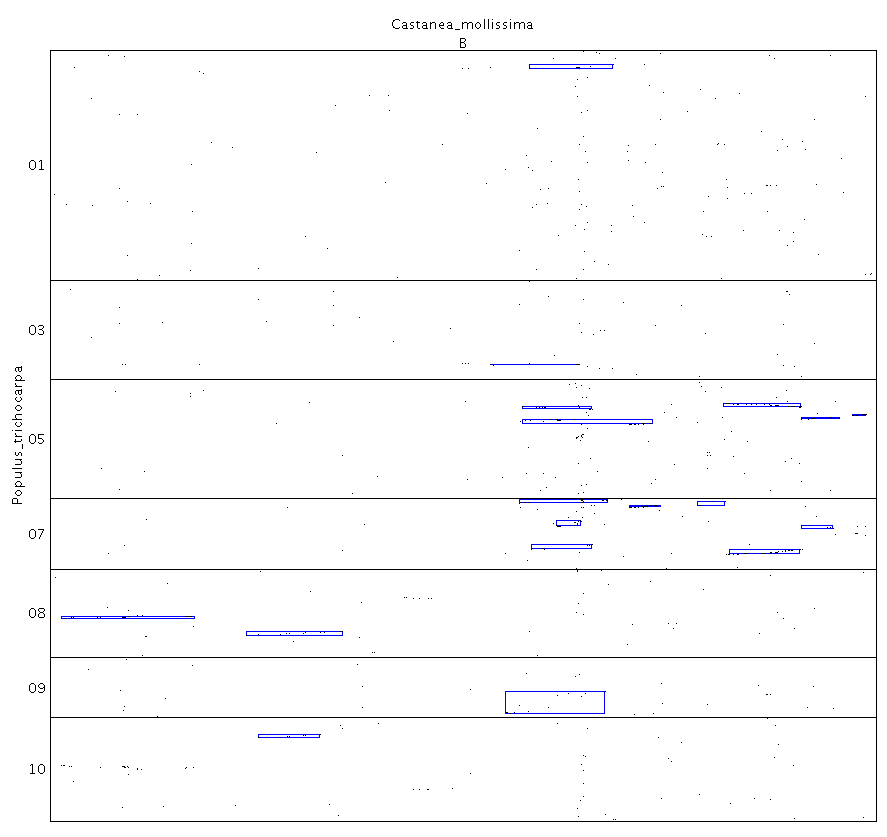


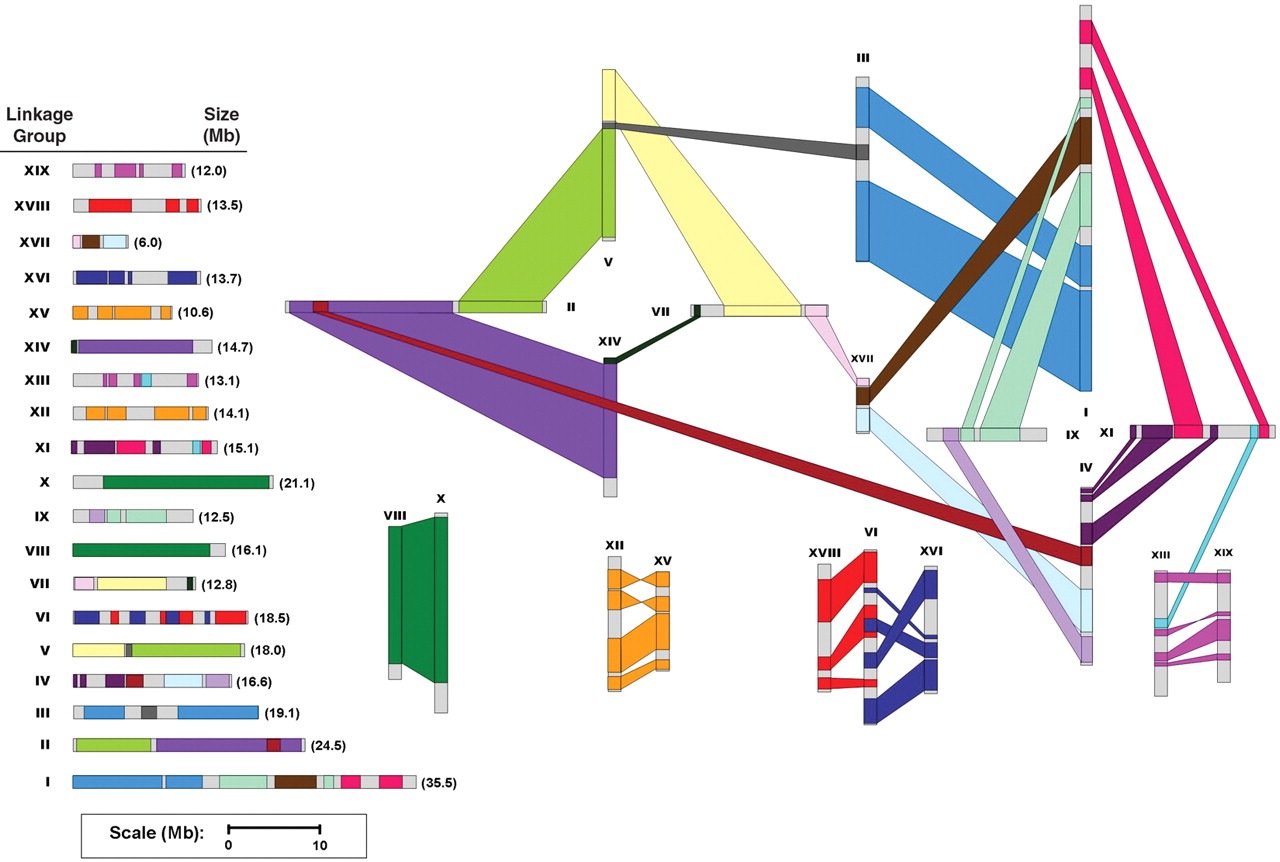


Chestnut Linkage group C. The overlap between populus LG V and XIII is not supported by the WGD analysis by Tuskan et al., 2006.


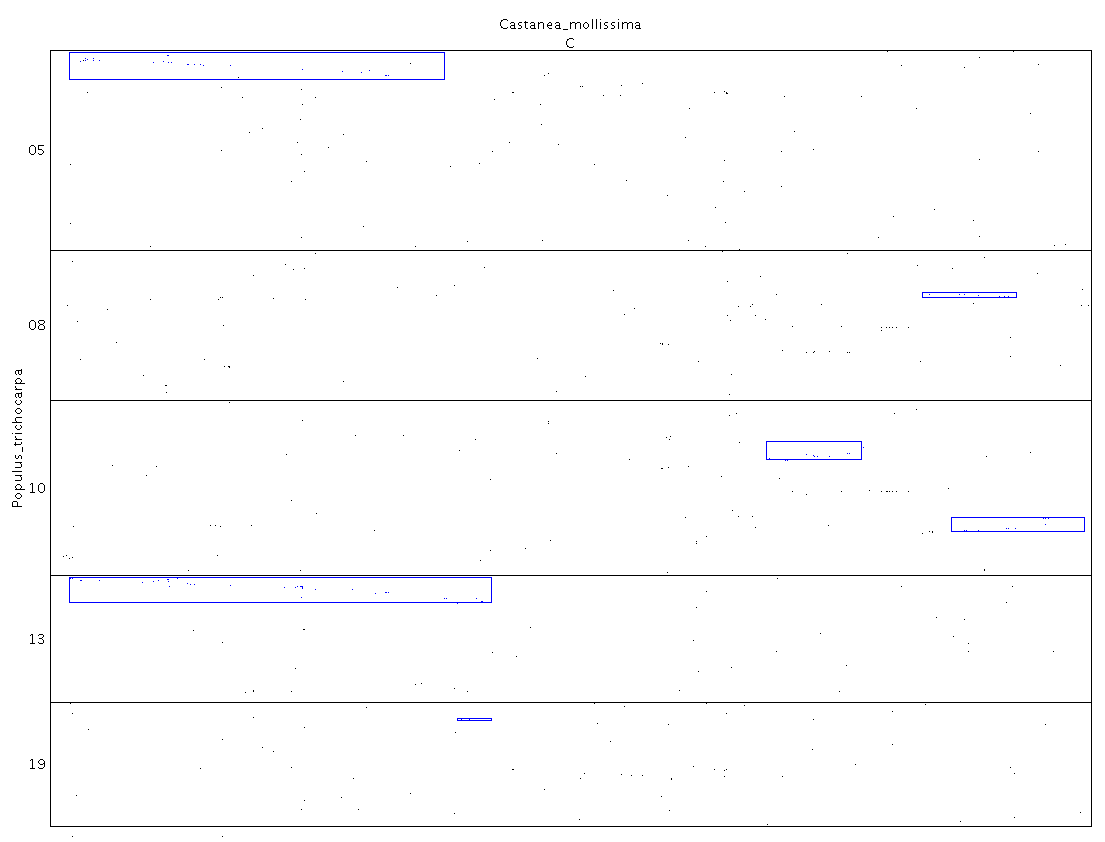


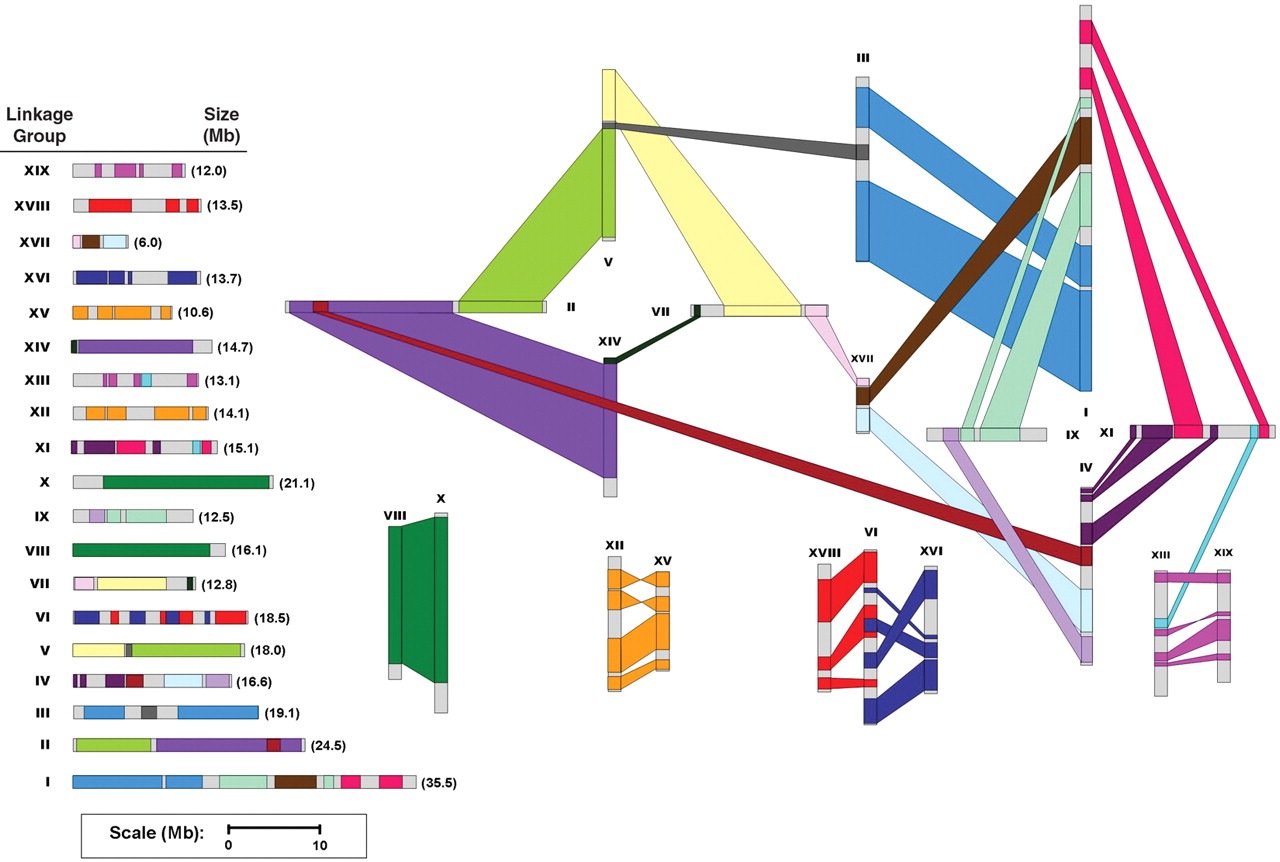


Chestnut Linkage group D. The block of matching to LG XIX that overlaps with LG XII and XV is not supported by the WGD event.


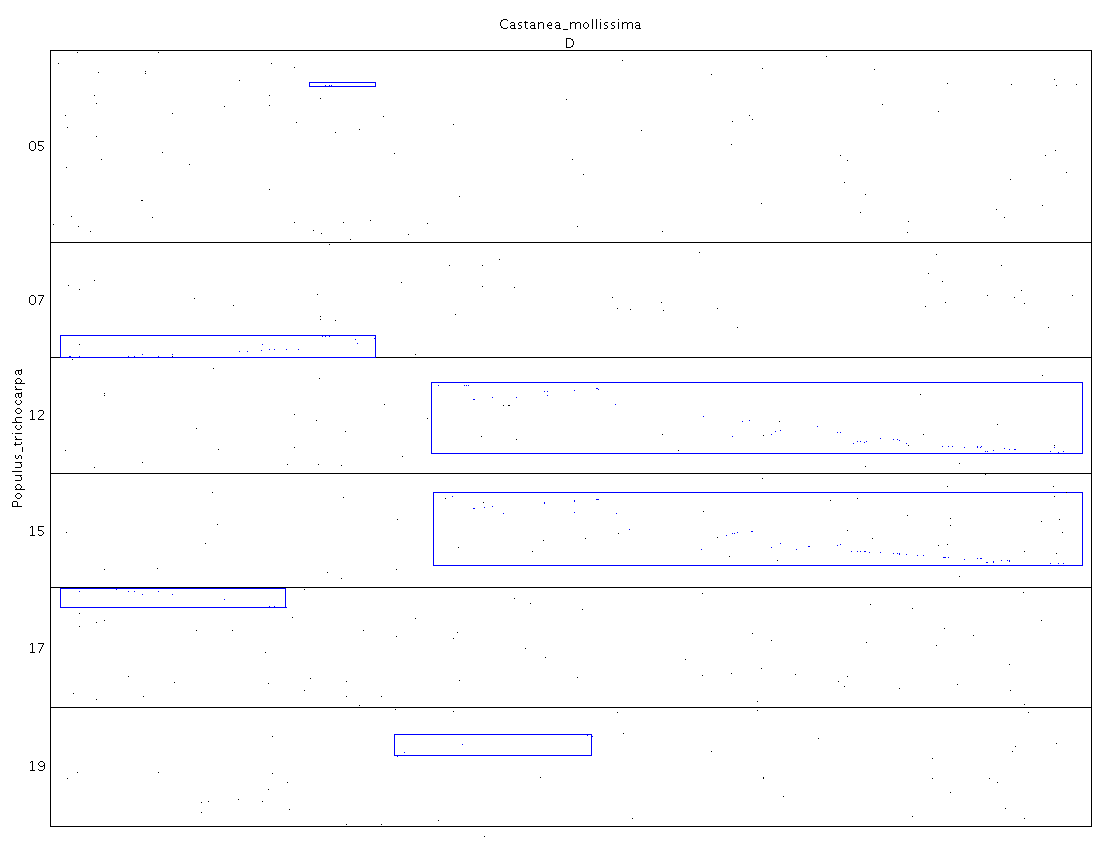

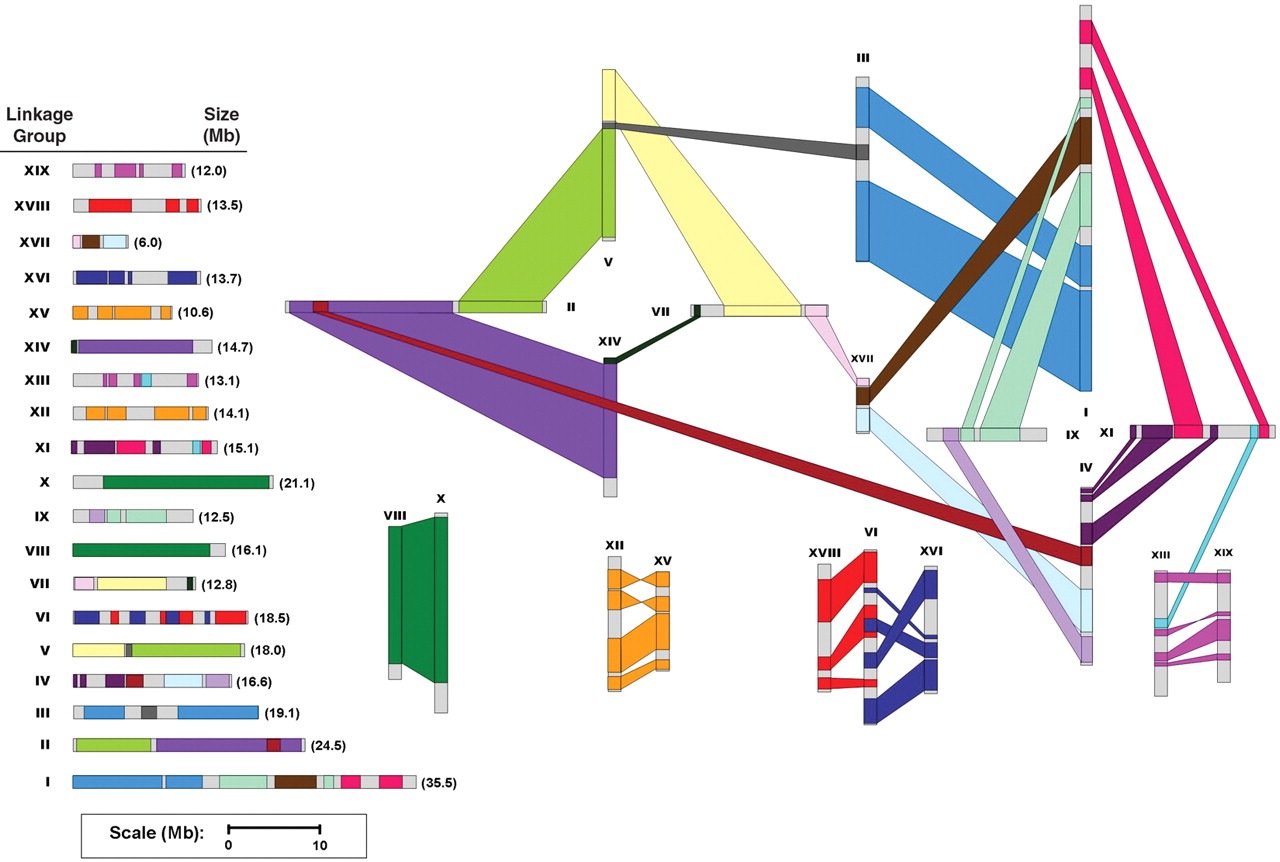


Chestnut Linkage group E.


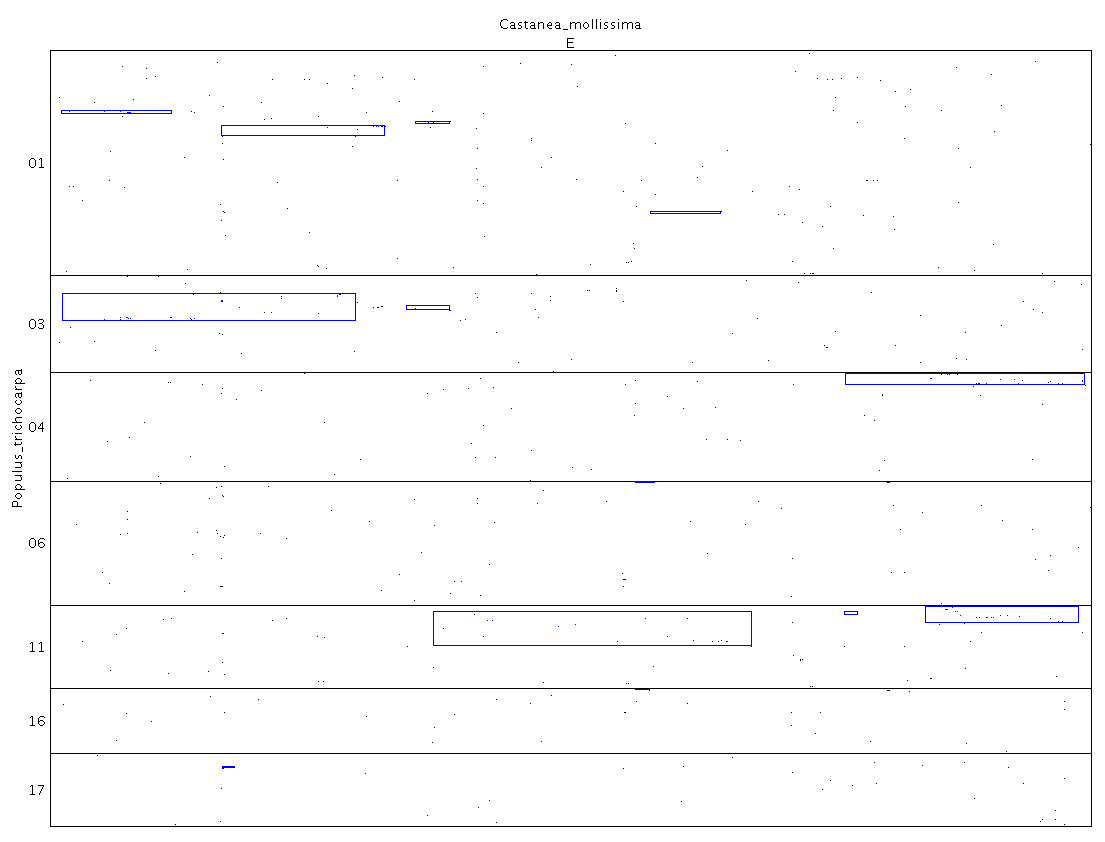


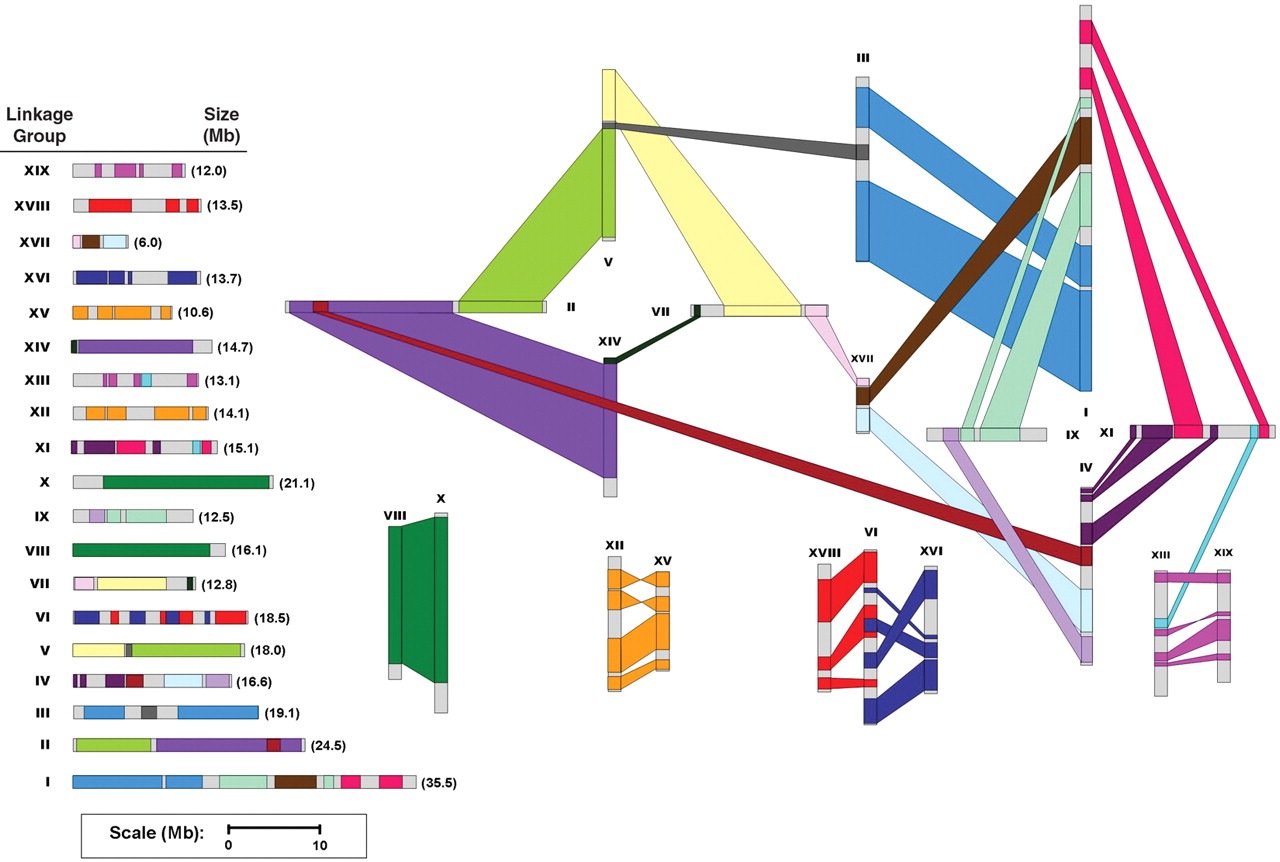


Chestnut Linkage group F. A large portion appears to overlap three populus chromosomes: I, XI and XIII. This is probably a combination of the overlapping portions of I to XI and a smaller segment of homology between XI to XIII.


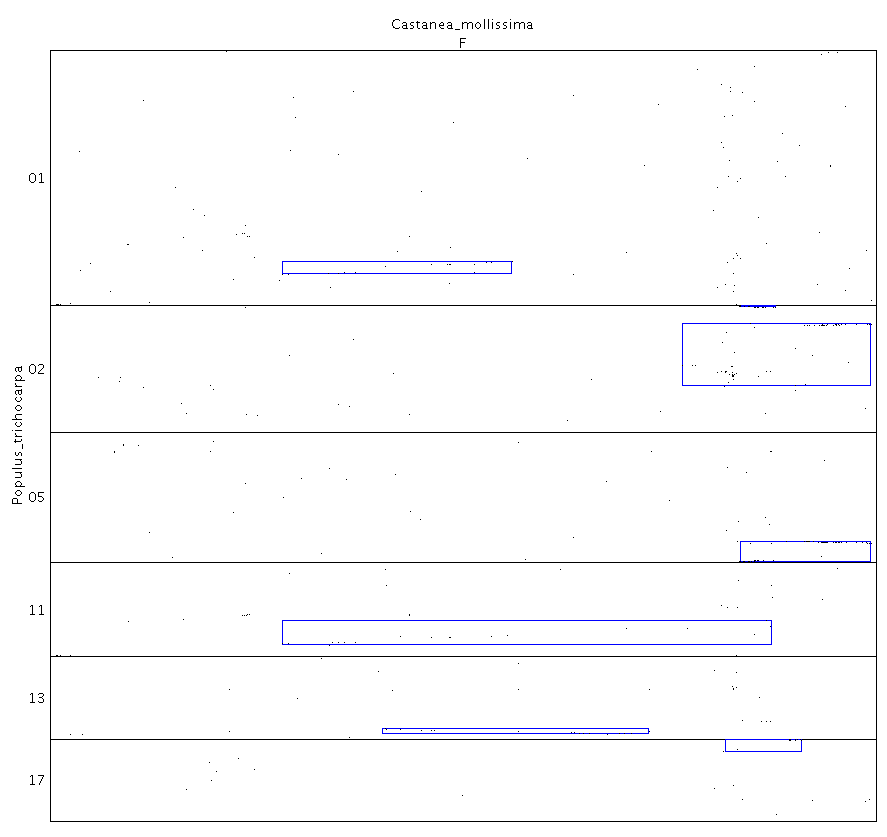


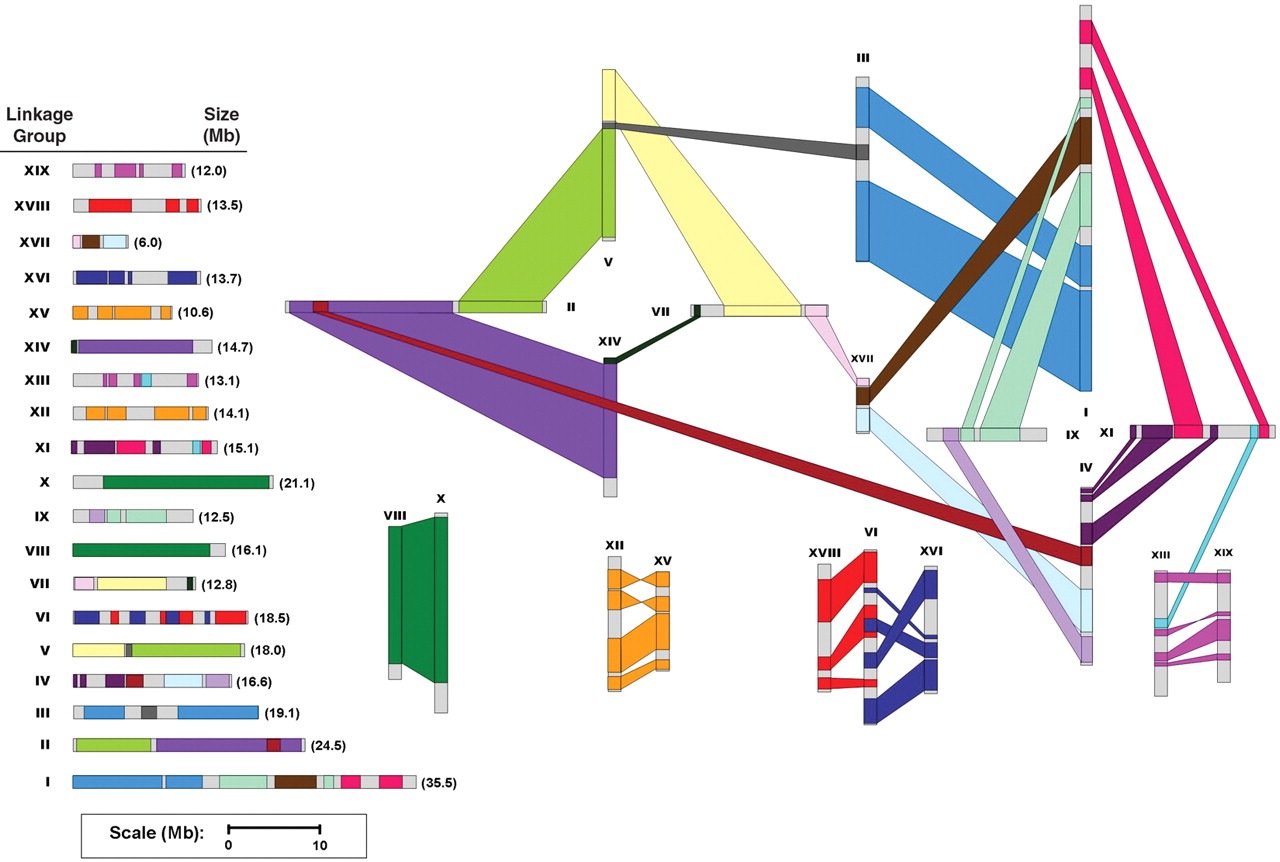


Chestnut Linkage group G.


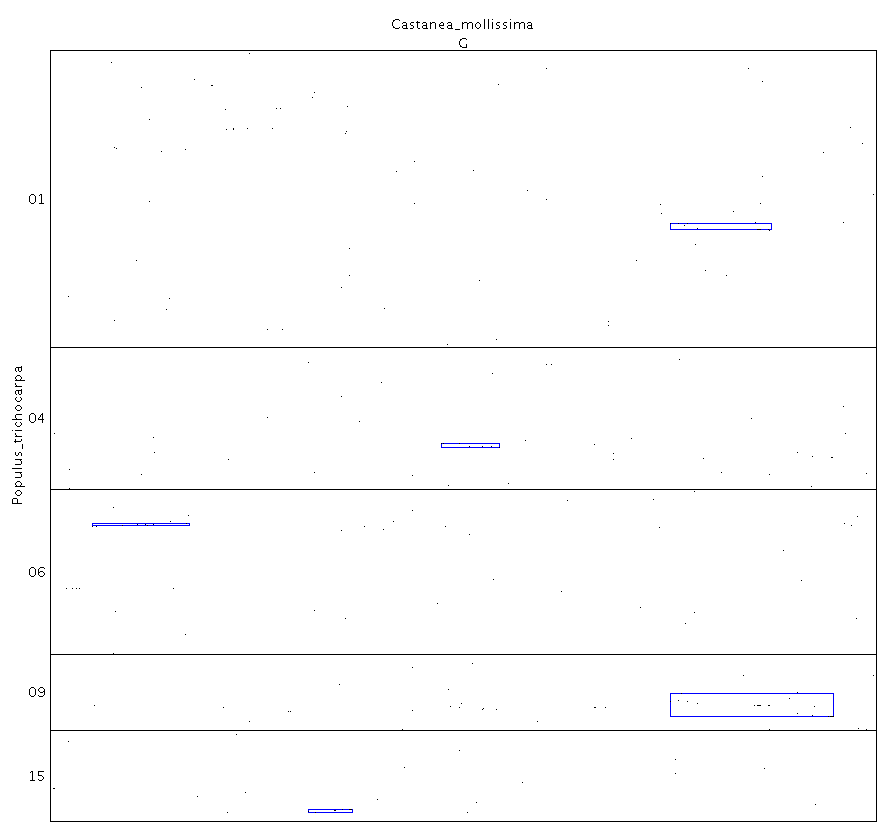


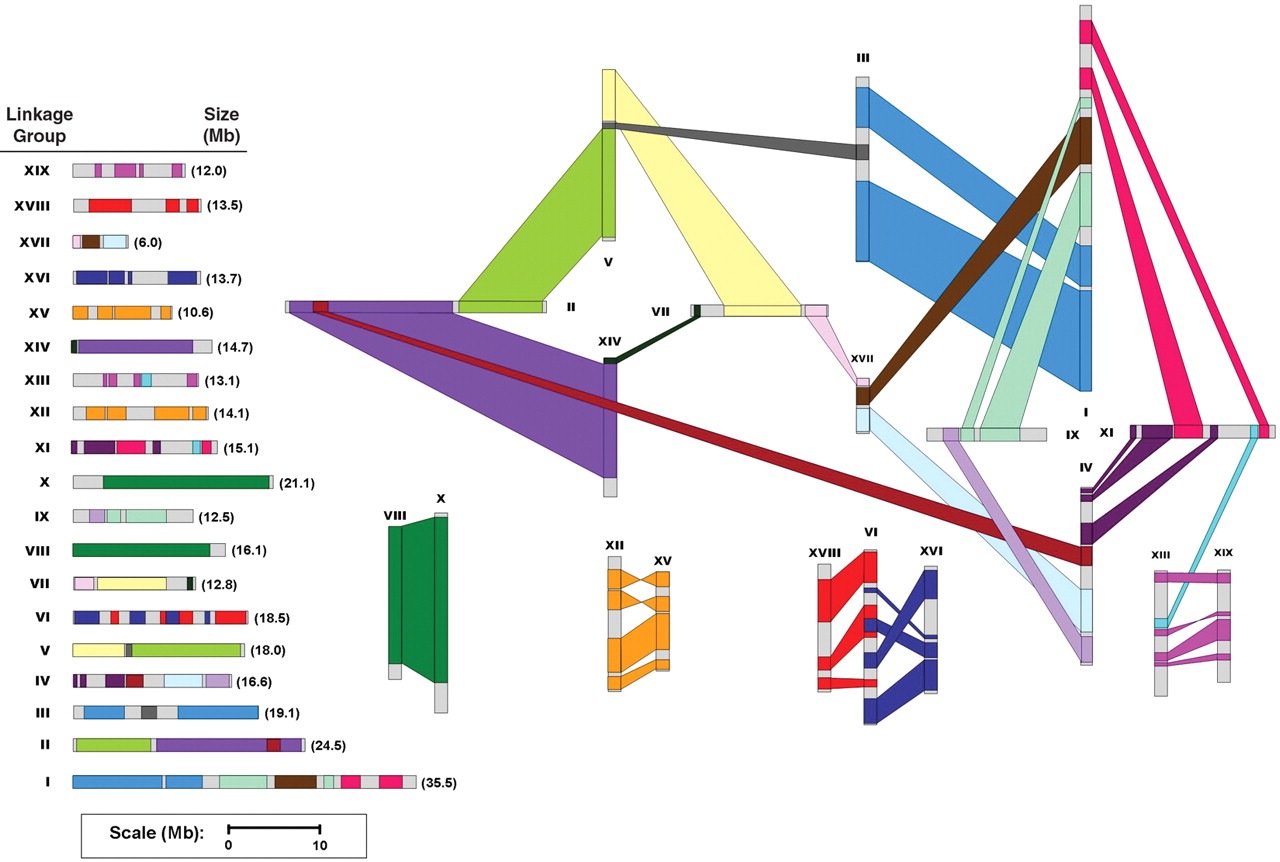


Chestnut Linkage group H.


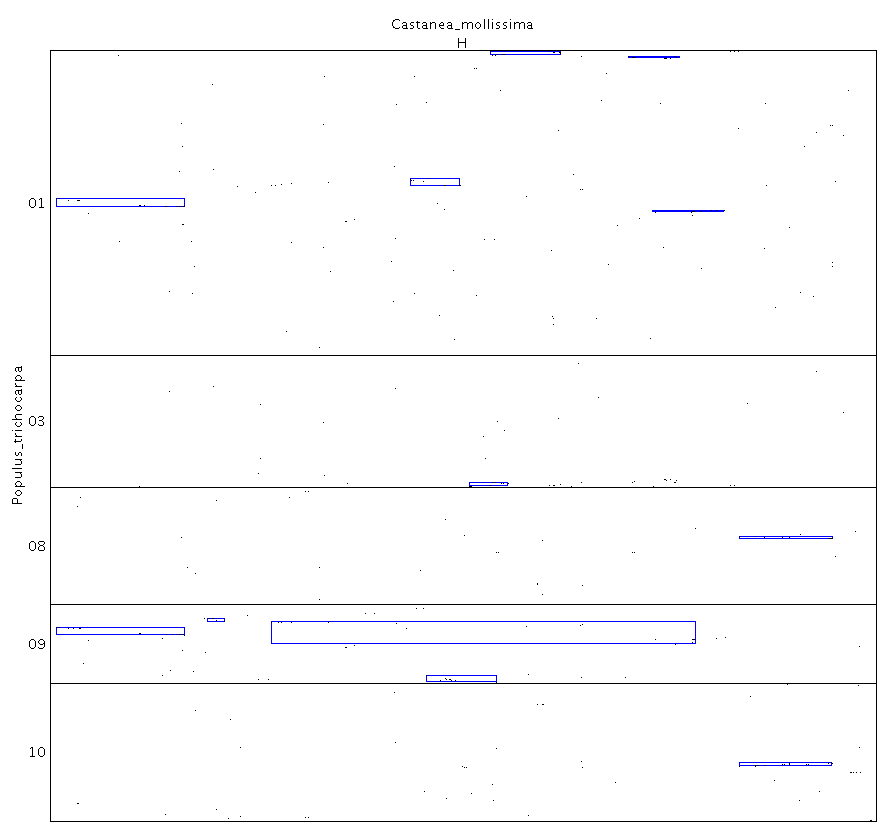


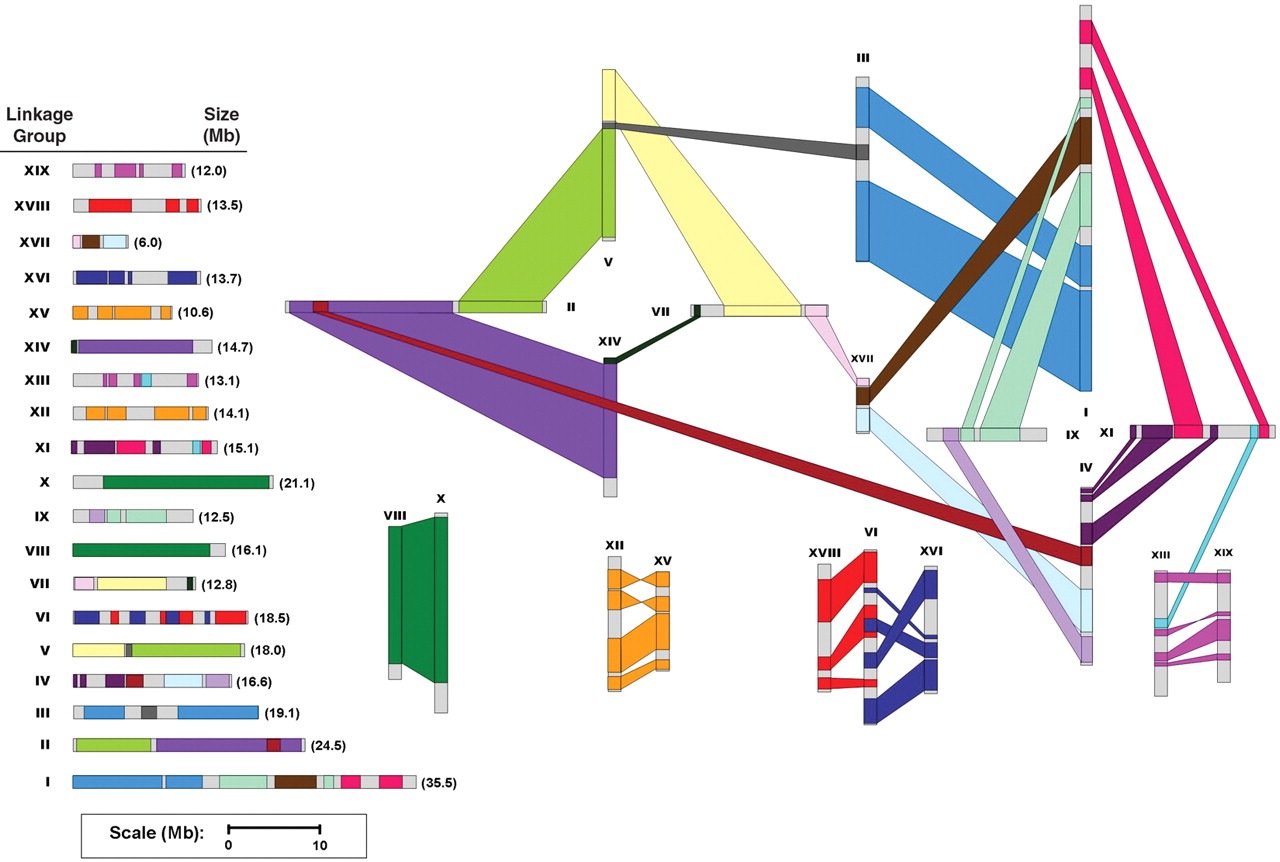


Chestnut Linkage group I.


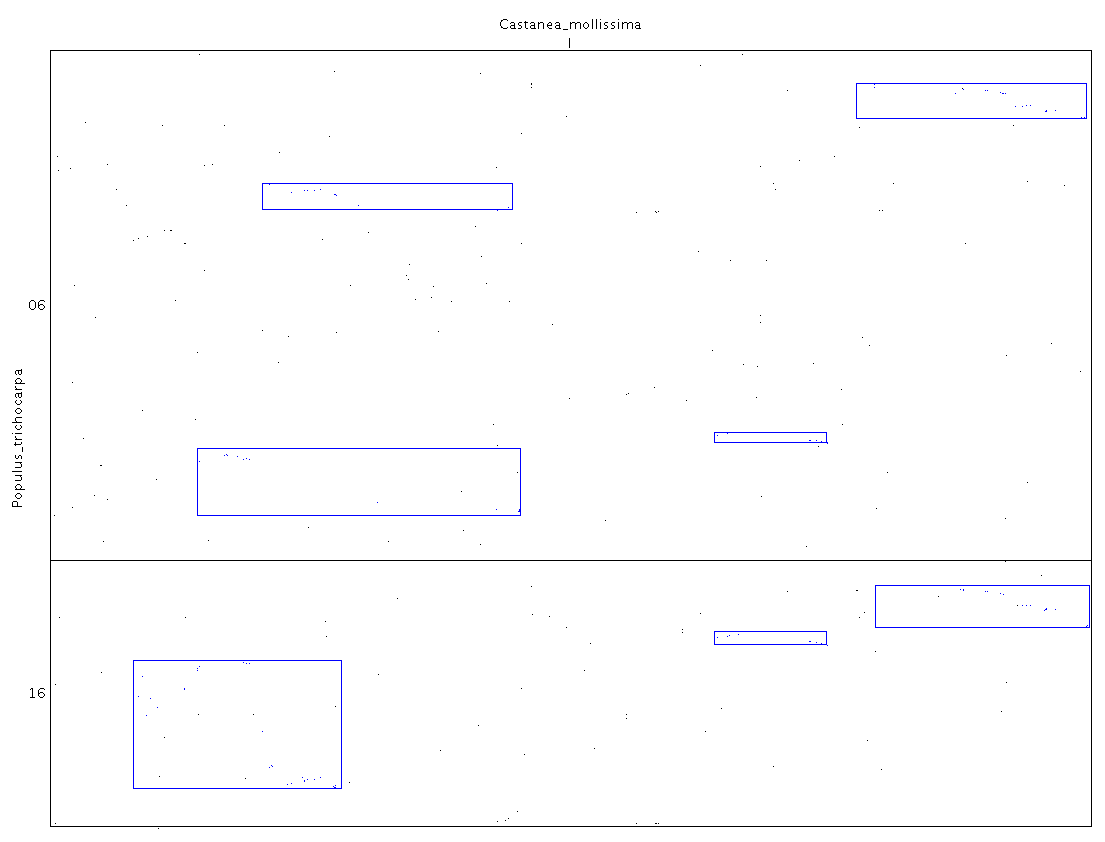


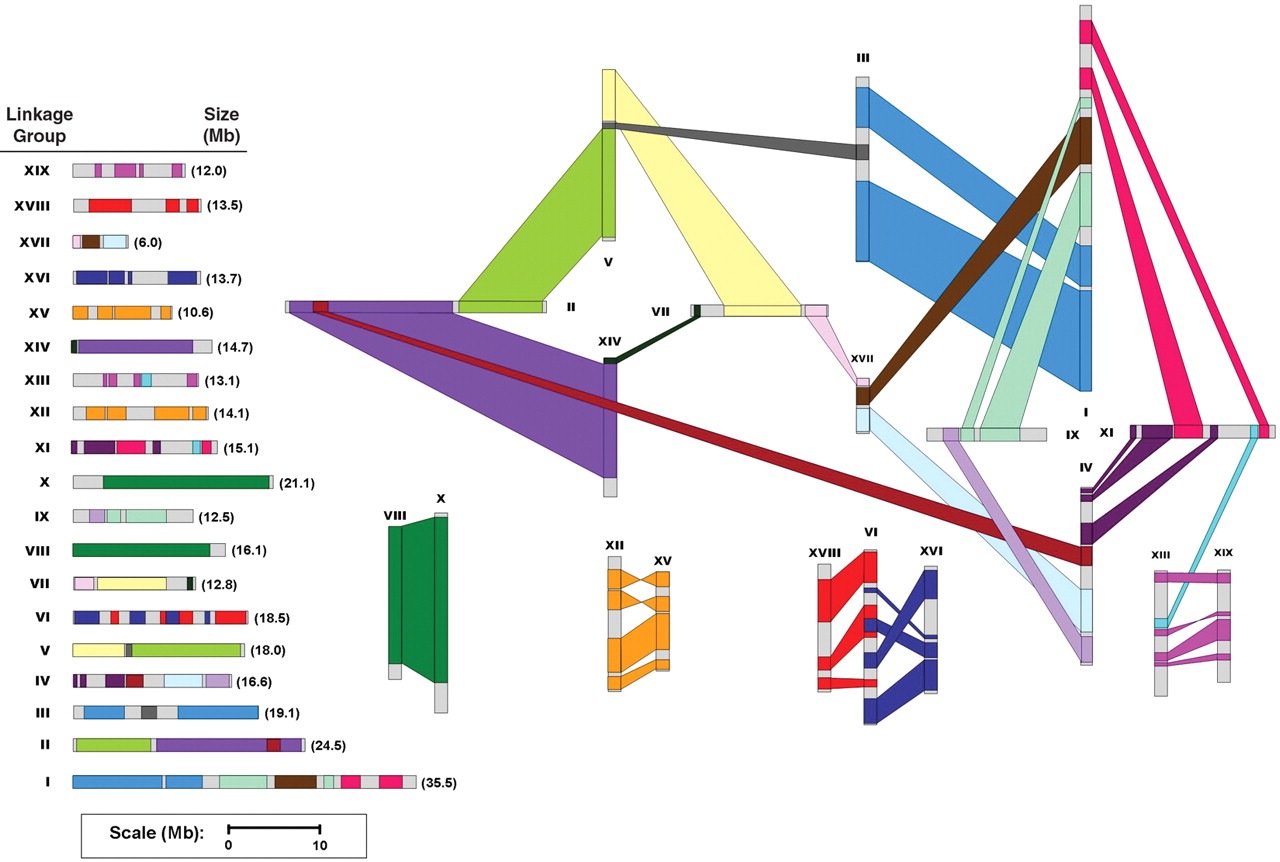


Chestnut Linkage group J. Homologous regions were found to populus chromosomes I and III and to chromosomes II and V.


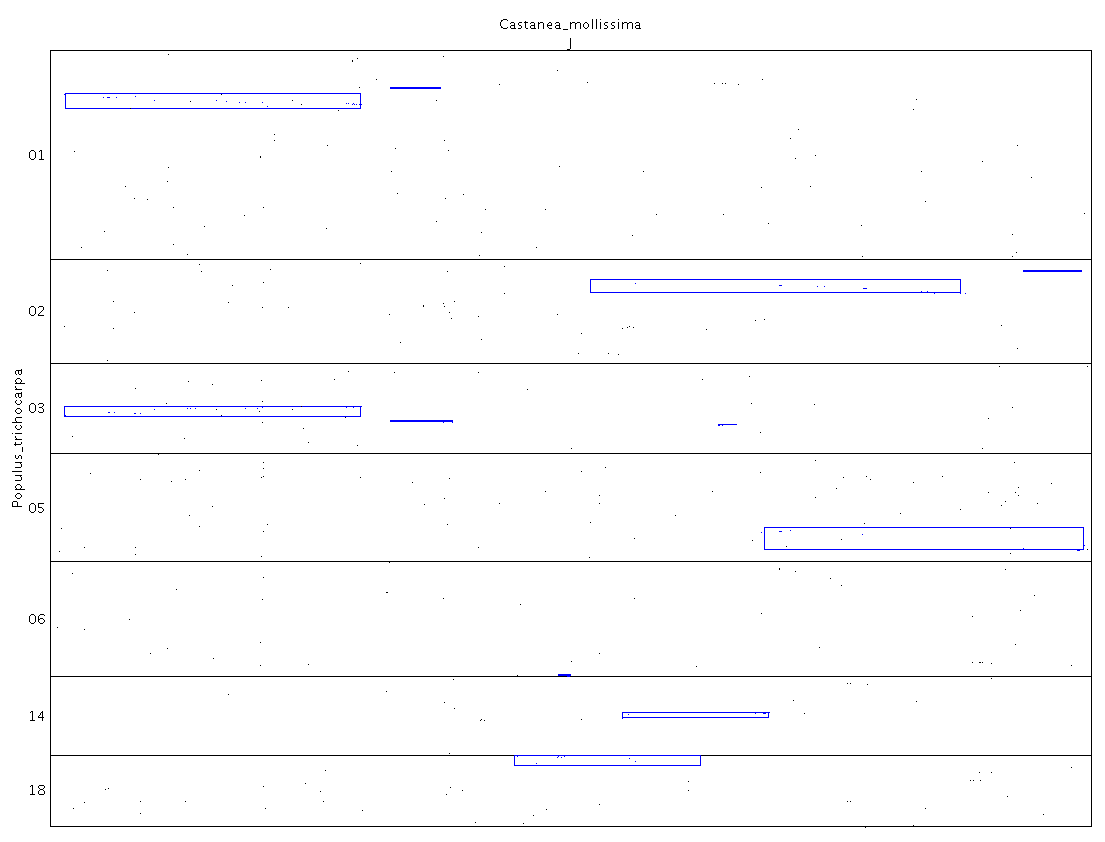


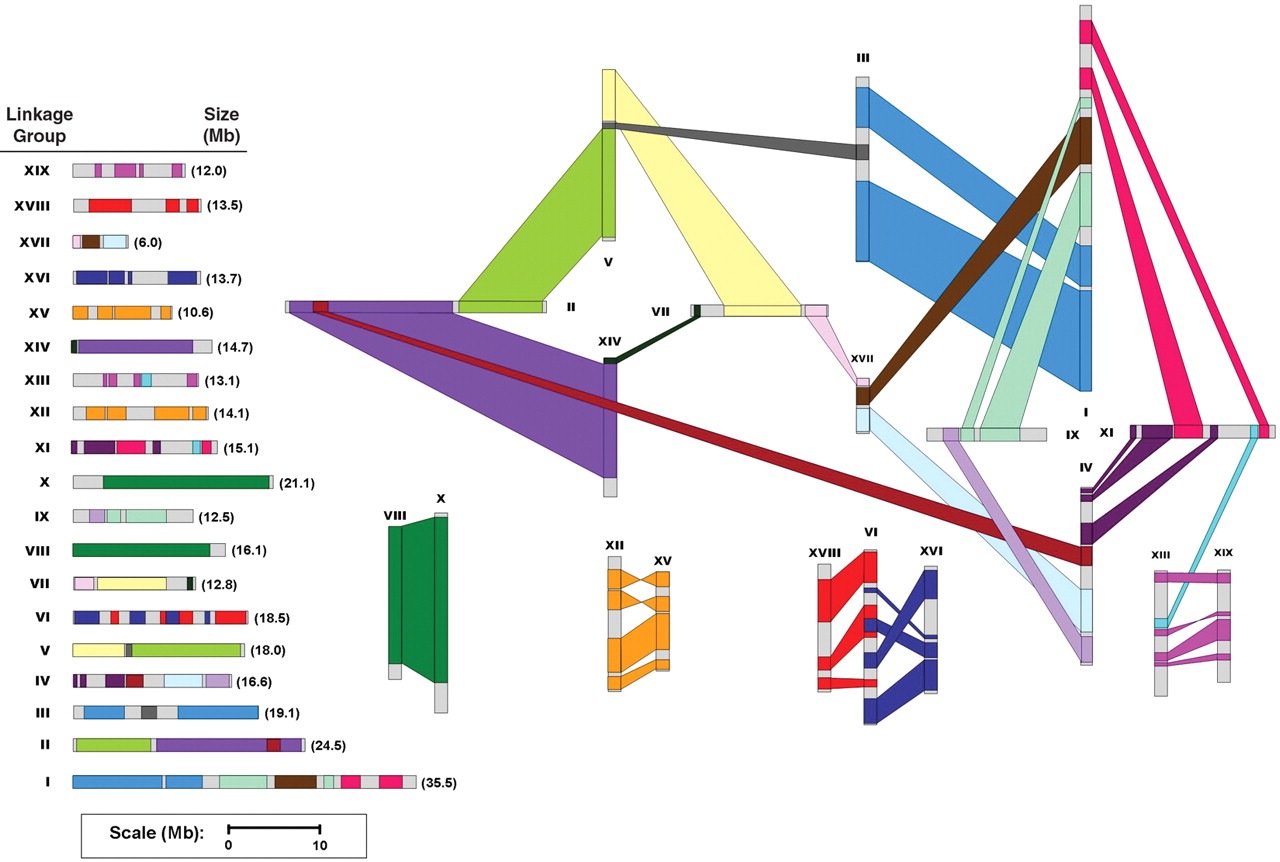


Chestnut Linkage Group K. Interestingly, a region appears to map to populus chromosomes II, VIII, X and XIV. There are known homologies between II and XIV and between VIII and X, but not across all four groups. One of the calculated syntenic regions has possibly been extended too far across LGK.


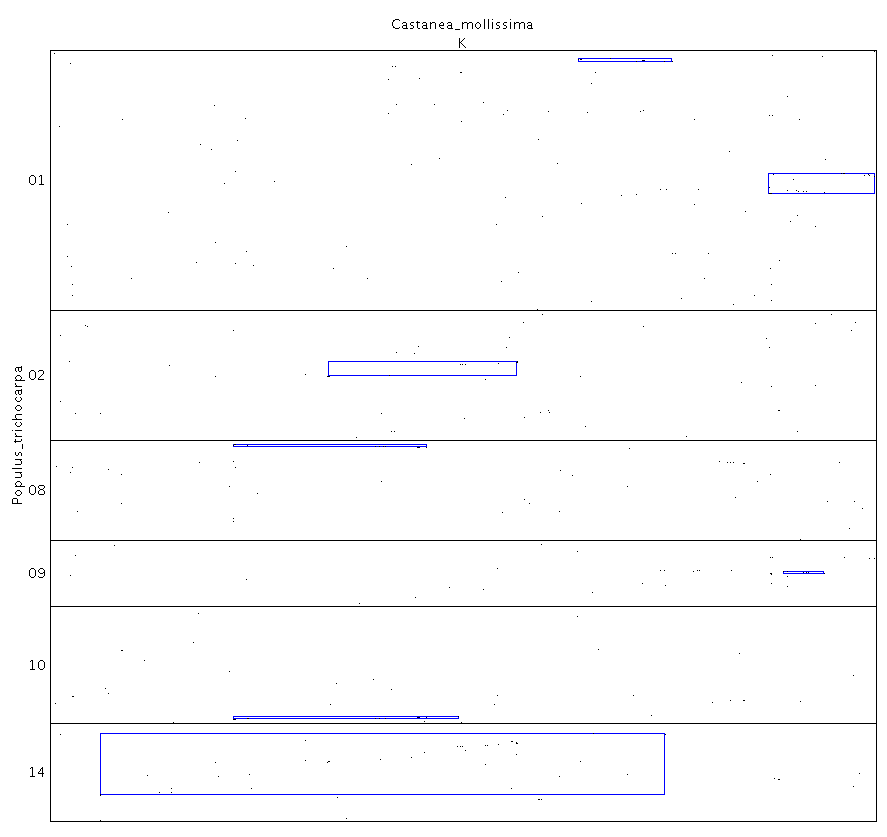


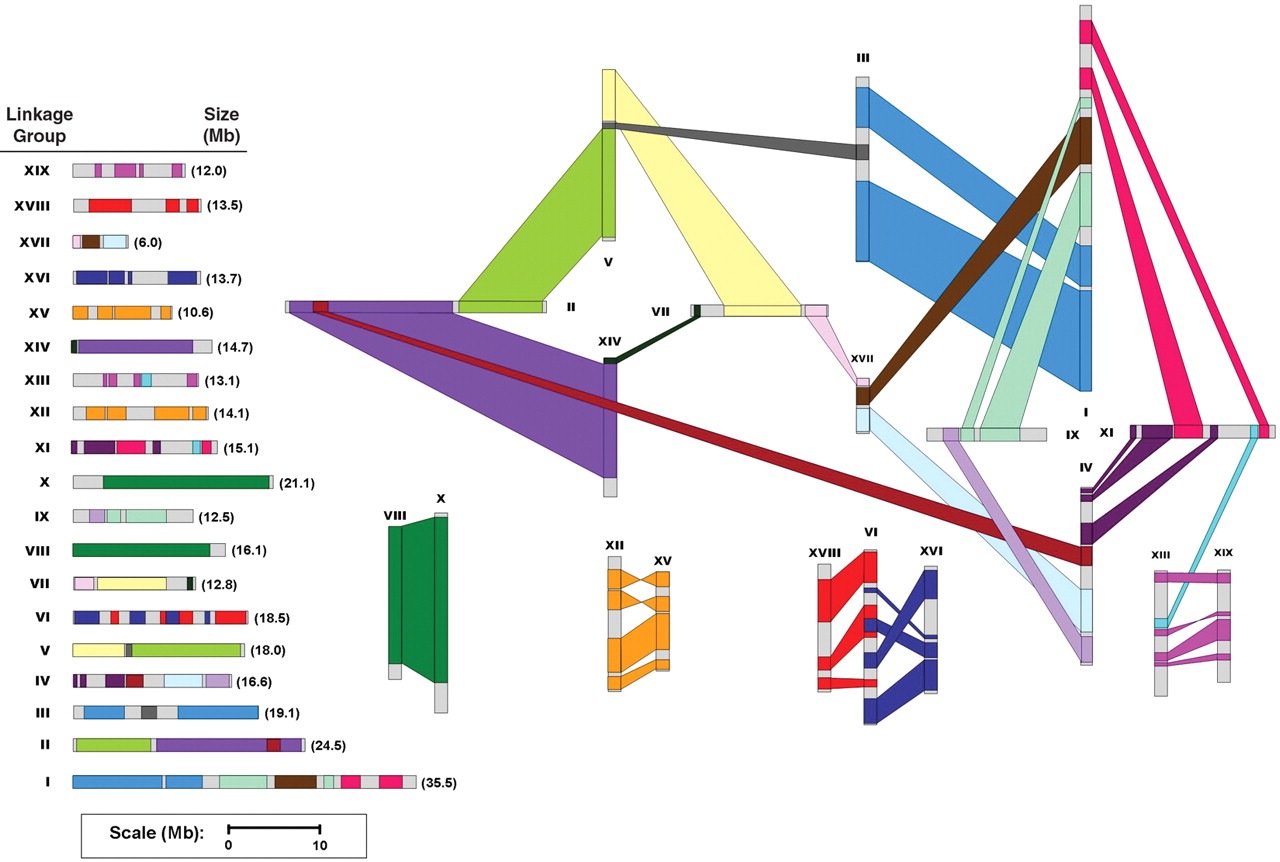


Chestnut Linkage group L.


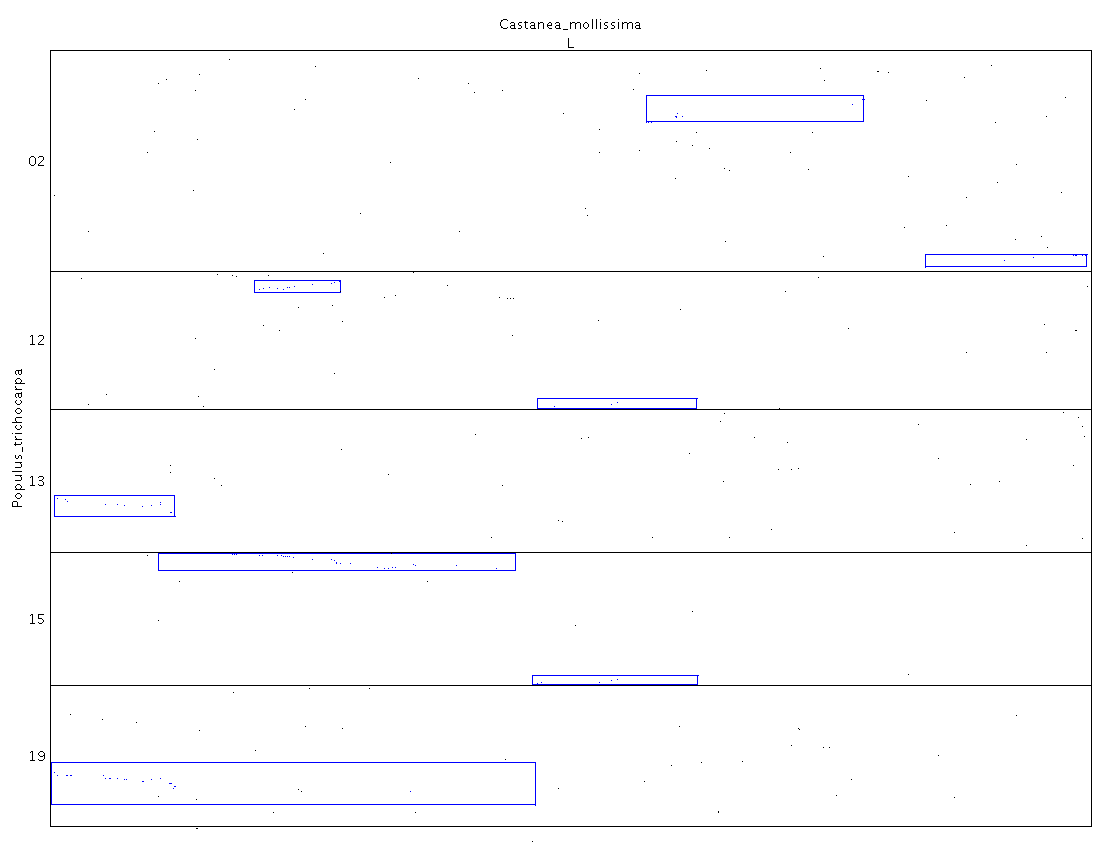


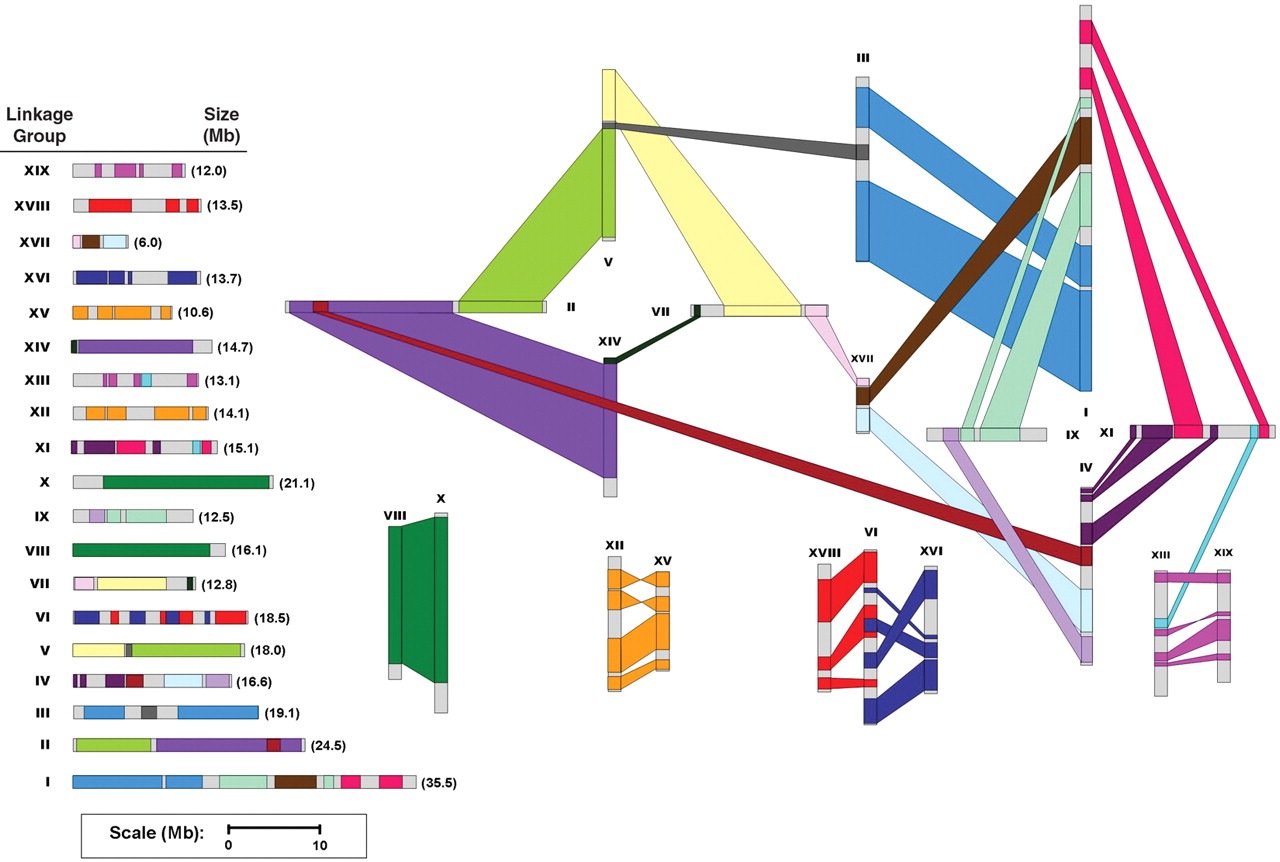

Supplement: Additional file 2: — Dot plots comparing each chestnut LG to the set of Populus trichocarpa chromosomes with highlightnig of areas of “double coverage” that correspond to the Populus WGD. (DOCX 808 kb) [file 12864_2015_1942_MOESM2_ESM.docx]
